# Latozinemab, a novel progranulin-elevating therapy for frontotemporal dementia

**DOI:** 10.1186/s12967-023-04251-y

**Published:** 2023-06-15

**Authors:** Michael Kurnellas, Ananya Mitra, Tina Schwabe, Robert Paul, Andrew E. Arrant, Erik D. Roberson, Michael Ward, Felix Yeh, Hua Long, Arnon Rosenthal

**Affiliations:** 1 Alector, Inc., 131 Oyster Point Blvd, #600, South San Francisco, CA 94080 USA; 2 Center for Neurodegeneration and Experimental Therapeutics, Alzheimer’s Disease Center, and Evelyn F. McKnight Brain Institute, Departments of Neurology and Neurobiology, University of Alabama at Birmingham, Birmingham, AL USA; 3 Present Address: Neuron23, South San Francisco, CA 94080 USA; 4 Present Address: Nine Square Therapeutics, Inc., South San Francisco, CA 94080 USA; 5 Present Address: Genentech, South San Francisco, CA 94080 USA

**Keywords:** Frontotemporal dementia, Latozinemab, Neuroprotection, Phase 1 clinical trial, Pharmacodynamics, Pharmacokinetics, Progranulin, Safety, Sortilin

## Abstract

**Background:**

Heterozygous loss-of-function mutations in the progranulin (PGRN) gene (*GRN*) cause a reduction in PGRN and lead to the development of frontotemporal dementia (FTD-*GRN*). PGRN is a secreted lysosomal chaperone, immune regulator, and neuronal survival factor that is shuttled to the lysosome through multiple receptors, including sortilin. Here, we report the characterization of latozinemab, a human monoclonal antibody that decreases the levels of sortilin, which is expressed on myeloid and neuronal cells and shuttles PGRN to the lysosome for degradation, and blocks its interaction with PGRN.

**Methods:**

In vitro characterization studies were first performed to assess the mechanism of action of latozinemab. After the in vitro studies, a series of in vivo studies were performed to assess the efficacy of a mouse-cross reactive anti-sortilin antibody and the pharmacokinetics, pharmacodynamics, and safety of latozinemab in nonhuman primates and humans.

**Results:**

In a mouse model of FTD-*GRN*, the rodent cross-reactive anti-sortilin antibody, S15JG, decreased total sortilin levels in white blood cell (WBC) lysates, restored PGRN to normal levels in plasma, and rescued a behavioral deficit. In cynomolgus monkeys, latozinemab decreased sortilin levels in WBCs and concomitantly increased plasma and cerebrospinal fluid (CSF) PGRN by 2- to threefold. Finally, in a first-in-human phase 1 clinical trial, a single infusion of latozinemab caused a reduction in WBC sortilin, tripled plasma PGRN and doubled CSF PGRN in healthy volunteers, and restored PGRN to physiological levels in asymptomatic *GRN* mutation carriers.

**Conclusions:**

These findings support the development of latozinemab for the treatment of FTD-*GRN* and other neurodegenerative diseases where elevation of PGRN may be beneficial.

*Trial registration* ClinicalTrials.gov, NCT03636204. Registered on 17 August 2018, https://clinicaltrials.gov/ct2/show/NCT03636204.

**Supplementary Information:**

The online version contains supplementary material available at 10.1186/s12967-023-04251-y.

## Background

Frontotemporal dementia (FTD) is a rare, early-onset form of dementia with an estimated prevalence of 4 to 22 per 100000 people aged younger than 64 years [1–3]. FTD is composed of several clinical phenotypes that manifest as changes in behavior, personality, language, cognitive skills, and motor function, and FTD is associated with synapse loss, gliosis, neuronal loss, and, ultimately, gross atrophy in the frontal and temporal lobes of the brain [2, 4–6]. The disease progresses rapidly and is associated with an estimated life expectancy of 7–9 years after symptom onset [7].

A family history of FTD is present in 20–40% of cases, and 10% of cases are inherited in an autosomal dominant pattern [8–11]. Most of the genetic component is accounted for by a mutation in 1 of 3 genes: progranulin (*GRN*), microtubule-associated protein tau, and chromosome 9 open reading frame 72 (*C9orf72*) [7]. *GRN* mutations have been identified in approximately 10–23% of people with a family history consistent with autosomal dominant FTD and in approximately 3% of patients with FTD of apparently sporadic origin [12]. More than 100 *GRN*-heterozygous loss-of-function (LOF) mutations have been identified [7, 13]. *GRN* mutations cause disease with a wide range of ages of onset and phenotypic variability, even within families [7, 13]. Though the clinical presentation and age of onset are impacted by additional genetic modifiers such as TMEM106B [14–17], the disease penetrance approximates 100%, making *GRN* mutations causal for FTD [18–20].

Because progression to dementia is practically certain in *GRN* mutation carriers, it is imperative to develop effective treatments for FTD. Progranulin (PGRN) levels are 50% to 70% below normal in the plasma and CSF of both affected patients and asymptomatic *GRN* mutation carriers (aFTD-*GRN*) [21–23]. Thus, restoring PGRN levels to a normal range may be an effective therapeutic approach for FTD caused by *GRN* LOF mutations (FTD-*GRN*).

Identified receptors for PGRN include sortilin [24, 25], ephrin type-A receptor 2 [26], notch [27], epidermal growth factor receptor [28], and mannose 6-phosphate receptor (M6PR) and low-density lipoprotein receptor–related protein 1 (LRP1), both of which bind PGRN in conjunction with the secreted protein prosaposin [29]. Sortilin binds extracellular PGRN and targets it for lysosomal degradation [25]. Polymorphic variants of the sortilin gene (*SORT1*) have been linked to PGRN levels in serum, as well as to FTD susceptibility [24, 30, 31]. Further, genetic ablation of *Sort1* in mice led to 2- to threefold higher levels of PGRN, and knockdown of *SORT1* in cultured HeLa cells has been shown to increase extracellular PGRN [24, 25]. We therefore hypothesized that a molecule that blocks the sortilin-PGRN interaction and/or decreases sortilin levels would mimic the genetic ablation of *Sort1*. Accordingly, these actions would be expected to increase the levels of residual PGRN in *GRN* mutation carriers, restore PGRN back to physiological levels, and slow or halt the progression of FTD-*GRN* [32].

Latozinemab (AL001) is a recombinant monoclonal antibody that binds specifically to sortilin, prevents sortilin-PGRN interactions, and facilitates a decrease in the levels of sortilin. Latozinemab increases the levels of PGRN in plasma and cerebrospinal fluid (CSF) and has the potential to slow disease progression in carriers of heterozygous LOF *GRN* mutations [32, 33], as well as in other neurodegenerative diseases where genetic mutations reduce PGRN levels, such as Parkinson’s disease [34, 35], Alzheimer’s disease [36], amyotrophic lateral sclerosis [37, 38], and limbic-predominant age-related TDP-43 encephalopathy [39].

Here, we report a comprehensive assessment from initial drug discovery to a first-in-human study, detailing the pharmacology, pharmacokinetics (PK), and pharmacodynamics (PD) of latozinemab. We first characterized the mechanism of action of latozinemab in vitro and then demonstrated target engagement of S15JG, a murine cross-reactive anti-sortilin antibody, and latozinemab in vivo in mouse and nonhuman primate studies, respectively. These preclinical studies led to a first-in-human phase 1 clinical trial designed to evaluate the safety, tolerability, PK, and PD of a single infusion of latozinemab in healthy volunteers (HVs) and aFTD-*GRN* participants.

## Methods

### Antibody generation

Latozinemab (AL001) is a human monoclonal immunoglobulin (IgG) G1m17,1 kappa antibody generated against human receptor sortilin and derived from human germlines IGHV4-B and IGKV2-28. Latozinemab was generated and affinity-matured through collaboration between Alector and Adimab (Lebanon, New Hampshire, USA) to optimize in vitro and in vivo activity and to ensure manufacturing development capability [40]. Three point mutations, L234 to A234, L235 to A235, and P331 to S331 (Kabat numbering system; L239L240 and P336 in sequential numbering), were made to the heavy chain to minimize effector functions, such as Fc gamma receptor binding, complement activation, and antibody-dependent cell-mediated cytotoxicity (ADCC) [41–43]. Reduction of Fc gamma receptor binding, complement activation, and ADCC have been verified by Alector. Minimization of the effector functions is a desired characteristic for latozinemab, which is intended for chronic administration to study participants and patients.

### In vitro cell-based assays of latozinemab

*Anti-sortilin antibody binding to human sortilin in HEK293T cells*. A stable HEK293T cell line expressing human sortilin was established by viral infection of a proprietary plasmid expressing human sortilin (GenScript Biotech, Piscataway, NJ). Cells were selected using hygromycin resistance, and a high-expressing monoclonal cell line was established. Parental HEK293T cells were utilized for control. For the binding assay, HEK293T cells were harvested by trypsinization, washed in PBS, and then counted and plated on 96-well plates at 1 × 10^5^ cells/well. The plates were spun at 1400 rpm for 3 min, and latozinemab or control antibodies were added in fluorescence-activated cell sorting (FACS) buffer (PBS + 2% fetal bovine serum [FBS]) and incubated on ice for 1 h. Cells were subsequently centrifuged as before and washed thrice with FACS buffer. Cells were then incubated with goat anti-human phycoerythrin (PE)–conjugated secondary antibody (1:100; Southern Biotech, Birmingham, AL; cat. #2040–09, lot #C1316-Q487) in FACS buffer for 30 min on ice. Cells were again washed thrice with FACS buffer and analyzed on an Intellicyt iQue® Flow Cytometer (Essen BioScience, Inc., formerly Intellicyt Corporation, part of the Sartorius Group; Göttingen, Germany). Binding was measured as mean fluorescence intensity (MFI) of PE.

*Cell-based competition assay for latozinemab antibody blocking of PGRN binding to sortilin*. Recombinant human PGRN (AdipoGen Inc., San Diego, CA) was biotinylated with an EZ-Link Micro NHS-PEG4 Biotinylation Kit from ThermoScientific™/Pierce™ (Waltham, MA) according to the manufacturer’s instructions. Sortilin-expressing HEK293T cells or control cells were harvested and washed in PBS. Biotinylated human PGRN at 20 nM was added in PBS + 2% FBS with or without latozinemab or isotype control human antibodies and incubated on ice for 2 h. After washing cells thrice in PBS + 2% FBS, cells were incubated in streptavidin-allophycocyanin (APC; 1:100; eBioscience, San Diego, CA; cat. #17-4317-82, lot #4303110) on ice for 30 min. The cells were then rewashed, resuspended in PBS + 2% FBS, and analyzed using an Intellicyt iQue Flow Cytometer. PGRN binding was measured as the MFI of APC of the sortilin-expressing cell population.

*Interaction between surface sortilin expression and PGRN levels in the presence of latozinemab*. Human U251 cells (American Type Culture Collection, Manassas, VA) have detectable cell surface expression of human sortilin and were utilized for measuring sortilin expression. U251 cells were plated at 3000 cells per well of a 96-well plate for 24 h, followed by incubation with latozinemab or isotype control antibodies for 48 h. Cells were harvested with trypsin, washed in PBS, and labeled with Dylight^™^-650 conjugated anti-sortilin antibody S2-11-650 (generated in-house). After cells were incubated with 5 μg/mL of the fluorescently conjugated sortilin antibody S2-11-650 for 1 h on ice, cells were washed thrice in PBS + 2% FBS and binding was quantified using an Intellicyt iQue Flow Cytometer as MFI of APC.

PGRN levels were quantified from the supernatants of the treated cells using an enzyme-linked immunosorbent assay (ELISA) for human PGRN, according to the manufacturer’s instructions (AdipoGen Life Sciences, Inc., San Diego, CA; cat. #AG-45A-0019YEK-KI01). Optical densities were measured at 450 nm using a BioTek Synergy HT Multi-Detection Microplate Reader (Winooski, VT). Data were analyzed using Microsoft Excel (Redmond, WA) and GraphPad Prism 9 (San Diego, CA).

### In vivo assessment of an anti-sortilin antibody in a mouse model of GRN haploinsufficiency

*Animals and treatment. Grn*^+/−^ mice and wild-type (WT) littermate controls of both sexes, age 19 to 21 months at the start of experiment, were used (N = 42: 21 were male and 21 were female; there were 9–12 in each of the 4 genotype/treatment groups). Mice were on a congenic C57BL6/J background and were housed in ventilated cages in a barrier facility with 12:12 light cycles and ad libitum access to food and water. All experiments were approved by the Institutional Animal Care and Use Committee of the University of Alabama at Birmingham.

Mice underwent pretreatment social dominance testing, in which WT and *Grn*^+/−^ mice were paired to confirm the presence of the expected low dominance phenotype in *Grn*^+/−^ mice. Treatment group assignment was stratified by winning percentage; both WT treatment groups had a mean pretreatment winning percentage of 67% vs *Grn*^+/−^ mice and both *Grn*^+/−^ treatment groups had a mean pretreatment winning percentage of 33% vs WT mice. Mice were treated with weekly intraperitoneal (i.p.) injections of S15JG or isotype control at 40 mg/kg for 4 to 5 weeks (totaling 5 injections prior to testing). S15JG is a mouse cross-reactive anti-sortilin antibody that was used in the mouse studies because latozinemab is not cross-reactive to rodents. Like latozinemab, S15JG binds to the beta-propellor region of sortilin (also the binding site for progranulin [25]) but at a slightly different epitope. Antibodies were coded so that the experimenter remained blind to treatment group during both injections and behavioral testing, with blind broken only after data analysis.

After treatment with S15JG, mice were retested for social dominance using the tube test, as previously described [44, 45] and detailed below. Following the behavioral studies, mice were administered antibodies for an additional 2 weeks before euthanasia, for a total duration of 7 weeks of antibody administration. Terminal bleeds were collected and plasma PGRN and white blood cell (WBC) sortilin levels were assayed, as described below.

*Tube test for social dominance*. Each mouse was tested against 3 mice from the opposing group in a 3-round design. Tests were designed so that mice from each group were evenly assigned to each side of the tube to avoid an effect of tube position. In cases where one group was paired against multiple other groups, rounds were interspersed to avoid an effect of testing order. Each mouse was limited to 6 rounds of testing per day to avoid testing fatigue. The winning percentage of each mouse (number of wins/total number of matches) was calculated and analyzed by Mann–Whitney test. All analyses were conducted with GraphPad Prism 9, with 2-tailed *p* values and α set at 0.05.

*Microdialysis sampling.* To evaluate the effects of an anti-sortilin antibody in the central nervous system (CNS), a separate group of mice were implanted in the medial prefrontal cortex with guide cannulas, and microdialysis sampling was performed after a single injection of S15JG or vehicle. Specifically, guide cannulas (Amuza, Inc., San Diego, CA) were implanted in the medial prefrontal cortex of *Grn*^+/−^ and WT mice 36 to 48 h before implantation of microdialysis probes. Cannulas were implanted using a stereotaxic frame (Stoelting Co., Wood Dale, IL) at 1.9 mm anterior to bregma, 0.4 mm lateral from the midline, and 1.0 mm below the surface of the skull. Mice were injected with either S15JG or control antibody (40 mg/kg, i.p.) 24 h before probe implantations and sampling. The antibodies were coded so that the experimenters were blind to treatment group until after sampling and analysis were complete. Microdialysis probes with 2-mm membranes (AtmosLM^™^, Amuza, Inc.) were implanted in the guide cannulas and perfused with artificial CSF (1.3 mM CaCl_2_, 1.2 mM MgSO_4_, 3 mM KCl, 0.4 mM KH_2_PO_4_, 25 mM NaHCO_3_, 122 mM NaCl, pH 7.35) at 1 μL/min. To optimize fluid recovery and prevent protein binding to tubing, artificial CSF was supplemented with bovine serum albumin (BSA) solution (30% BSA, MilliporeSigma, Burlington, MA) at a final concentration of 4% [46, 47]. Probes were perfused using a push–pull system [48] consisting of a syringe pump (Amuza, Inc.) and a peristaltic pump (Amuza, Inc.). Based on preliminary experiments, collection of samples was delayed for 8 h after probe implantation to allow stabilization of PGRN levels in interstitial fluid (ISF). Samples were then collected in a refrigerated fraction collector (FC-90; Amuza, Inc.) at 1-h intervals for 16 h, so that samples were collected from 32 to 48 h after antibody injection. After collection was completed, ISF samples were stored at − 80 °C until analysis by ELISA.

### PK/PD of latozinemab in nonhuman primates

*Subjects*. Twelve male cynomolgus monkeys (*Macaca fascicularis*), ranging from 31 to 41 months old, were used as subjects. All procedures complied with the Animal Welfare Act, the Guide for the Care and Use of Laboratory Animals, and the Office of Laboratory Animal Welfare.

*Study design*. Latozinemab was administered once via intravenous (i.v.) (bolus) injection on day 1 at a dose of 0 mg/kg (placebo), 5 mg/kg, 20 mg/kg, 60 mg/kg, or 200 mg/kg. Blood samples (1 mL) were collected from the femoral vein on day 1 at pre dose, and again at approximately 0.17, 0.5, 1, 2, 4, 6, 8, 12, 24, 36, 48, 72, 96, 144, 216, 312, 480, 648, 816, and 984 h post dose for plasma and WBCs. CSF samples (0.2 mL) were collected on day 1 at pre dose, and again at approximately 0.5, 1, 2, 4, 6, 8, 12, 24, 36, 48, 72, 96, 144, 216, 312, 480, 648, 816, and 984 h post dose. ELISA was used to measure concentrations of latozinemab and PGRN, as well as WBC expression of sortilin for PK and PD analyses.

### First-in-human phase 1 study

*Participants*. Participants were eligible for inclusion if they met the following key criteria: males or nonpregnant females; aged 18–65 years in the HV single ascending dose (SAD) groups or 18–80 years in the aFTD-*GRN* group; in good physical health on the basis of no clinically significant findings; and aFTD-*GRN* individuals who knew their *GRN* mutation status. Participants were excluded from the study if they met any of the following criteria, among others: known history of reactions to antibodies or fusion proteins; positive drug or alcohol results at screening; history of alcohol or substance abuse within the past 2 years; history of seizures, major depression, schizophrenia, schizoaffective disorder, bipolar disorder, or dementia due to a condition other than FTD; history of cancer with exceptions; history of severe, clinically significant CNS trauma; or any other severe or unstable medical condition or abnormal clinical laboratory test.

*Study design*. Study AL001-1 (NCT03636204) was a multisite, first-in-human phase 1 study designed to evaluate the safety, tolerability, PK, and PD of latozinemab administered as a single i.v. dose in HVs or aFTD-*GRN* participants. Safety, tolerability, PK, and PD of latozinemab administered as multiple doses (MDs) were also assessed in a cohort of symptomatic carriers of *GRN* mutations causative of FTD (data not shown for MD cohort).

In the HV SAD groups, participants were randomized 6:2 to latozinemab or placebo in 5 dose groups, including 2, 6, 15, 30, and 60 mg/kg. The first HV SAD group was dosed at 2 mg/kg, with doses escalating up to 60 mg/kg in subsequent groups. The group of aFTD-*GRN* participants (n = 6) was dosed at 60 mg/kg latozinemab after dose escalation was complete. On day 1, all participants received a single dose of their assigned treatment. During the follow-up period, participants returned for scheduled visits on days 2, 3, 6, 8, 13, 18, 25, 30, 43, 57, 85, and 113. Safety assessments were made at all visits. Serum PK samples, PGRN plasma samples, and whole blood for WBCs were collected at predefined timepoints on days 1, 2, 3, 6, 8, 13, 18, 30, 43, 57, 85, and 113. CSF samples were collected from all participants except HVs in the 2- and 6-mg/kg dose groups on days-1 (the day prior to dosing), 2, and 18.

*Study endpoints*. The safety endpoints included the incidence, type, and severity of adverse events (AEs), and the incidence of dose-limiting AEs (DLAEs) or AEs that led to study discontinuation. Additional safety endpoints included changes from baseline in clinical laboratory tests, electrocardiogram (ECG) assessments, vital signs, or antidrug antibodies, and any physical or neurologic abnormalities. PK endpoints included serum and CSF concentrations of latozinemab at specified timepoints, and PK parameters, including CSF:serum partition coefficients. PD endpoints assessed changes in the levels of PGRN in plasma and CSF after dosing relative to baseline concentration, and changes in sortilin expression in WBCs after dosing relative to baseline.

*Statistics*. For categorical variables, frequencies and percentages were presented. Continuous variables were summarized using descriptive statistics. For PK parameters, geometric mean and 95% confidence intervals were also presented. Unless otherwise specified, baseline was defined as the last non-missing assessment, including repeated and unscheduled measurements, prior to the start of study drug administration.

The mean concentration per timepoint for each dose group was summarized for serum and CSF PK samples. All serum and CSF concentration values below the limit of quantification (BLQ) were set to 0. Partition coefficients were calculated for each timepoint as the ratio of latozinemab CSF concentration to latozinemab serum concentration.

Descriptive statistics were used to summarize the PD endpoints. PGRN plasma concentrations and sortilin WBC concentrations that were BLQ were set to half the lower limit of quantification (LLOQ). Serum latozinemab concentration vs time was used to derive PK parameters by noncompartmental methods. PK parameters were summarized for the PK population using descriptive statistics.

*Study approval.* The study was conducted in accordance with the general principles set forth in the international Ethical Guidelines for Biomedical Research Involving Human Subjects, International Council for Harmonisation Guideline for Good Clinical Practice, and the Declaration of Helsinki. The sponsor ensured the study complied with all local, federal, or country regulatory requirements as applicable.

The protocol and informed consent form were reviewed and approved by the institutional review board (IRB)/independent ethics committee (IEC) of each participating clinical site prior to study initiation. All serious AEs (SAEs), regardless of causality, were reported to the IRB/IEC. Participants provided informed consent prior to study enrollment.

### Bioanalytical and PD procedures

For the detection of latozinemab concentrations, recombinant human sortilin was diluted in coating buffer in 96-well ELISA plates and incubated at 2–8 °C for 1 day. Diluted plasma (monkey), serum (human), or CSF (monkey or human) samples were added to the wells for 1–2 h at room temperature, followed by detection with horseradish peroxidase (HRP) conjugated goat antihuman IgG H + L monkey-adsorbed (monkey plasma or CSF), or biotinylated anti-AL001 with streptavidin-HRP A (human serum), or Pierce High Sensitivity Streptavidin-HRP (human CSF). Antibody PK parameters were calculated using Phoenix WinNonlin software.

Concentrations of PGRN were quantified in plasma (all species), CSF (monkey and human), and ISF (mouse). PGRN levels were measured by ELISA following the manufacturer’s instructions for samples collected from mice (R&D Systems; cat. #DY2557) and monkeys (AdipoGen; cat. #AG-45A-0019YEK-KI01, lot #K2701611). For detection of PGRN in human, biological samples were diluted (1:40 for plasma and 1:4 for CSF) in ELISA buffer, added to the plate, incubated for 1 h at 37 °C, and then the plate was incubated with detection antibody and HRP-labeled streptavidin solution followed by a tetramethylbenzidine (TMB) substrate solution, with washes in between each incubation. PGRN levels in ISF samples were analyzed by ELISA (AdipoGen). Prior to analysis, ISF samples were diluted 1:1 with ELISA buffer, and then loaded onto ELISA plates and analyzed according to the manufacturer’s instructions. Absorbance was read on a Biotek Synergy LX plate reader. PGRN was quantitated relative to a standard curve run on each plate. Data were analyzed with GraphPad Prism 9.

To process WBCs from mouse and monkey samples, 1 mL of ammonium-chloride-potassium (ACK) lysis buffer (Lonza) was added to 200 μL of whole blood, and then mixed and incubated on ice for 10 min. The sample was centrifuged at 500 *x*g for 10 min at 4 °C. The supernatant was aspirated and the pellet was resuspended in 1 mL ACK lysis buffer for 5 min on ice. The sample was recentrifuged at 500 *x*g for 10 min and the buffer was aspirated. The cycle was repeated until all red blood cells were lysed. For sortilin concentration, high-binding 96-well ELISA plates were coated with anti-sortilin antibody (S2-11; 1 μg/mL) in PBS at 4 °C overnight. Diluted WBC samples were added to the wells for 1 h at room temperature. Wells were then incubated with goat anti-human sortilin-biotinylated secondary antibody (0.1 μg/mL; cat. #BAF3154, lot #WTJ016011; R&D Systems) for 1 h followed by streptavidin HRP (cat. #DY998, lot #P104876; R&D Systems) for 20 min. Pierce 1-Step Ultra TMB was used for visualization and the reaction was stopped with 2N sulfuric acid. For mouse and monkey studies, optical densities were measured at 450 nm using a BioTek Synergy HT Multi-Detection Microplate Reader. Data were analyzed using Excel and GraphPad Prism 9.

The concentration of sortilin in human WBCs was assessed as follows. The S2-11 human IgG1 was diluted in coating buffer, added to the plate, and incubated overnight. The plate was washed and un-adsorbed sites were blocked with blocking buffer for 1–3 h. After washing the plate, lysed WBCs were diluted, dispensed onto the same plate, and incubated for 1 h. Then the plate was incubated with goat antihuman sortilin biotin, streptavidin-HRP A, and finally a working substrate solution, with washes in between each incubation.

## Results

### Latozinemab effectively binds sortilin with high affinity and blocks the interaction between PGRN and sortilin in vitro

Anti-sortilin antibodies were developed and tested for their ability to competitively block binding to PGRN in a cell-free assay. Blocking antibodies were further screened for their ability to induce sortilin degradation in a cell-based assay, and antibodies that displayed dual functionality of competitive blockade and receptor degradation were selected for antibody engineering and further studies. A lead human antibody was affinity-matured and engineered with an inert IgG1 containing L234A/L235A/P331S mutations of the heavy chain to minimize Fc receptor effector functions, and was subsequently termed latozinemab.

**Fig. 1:**
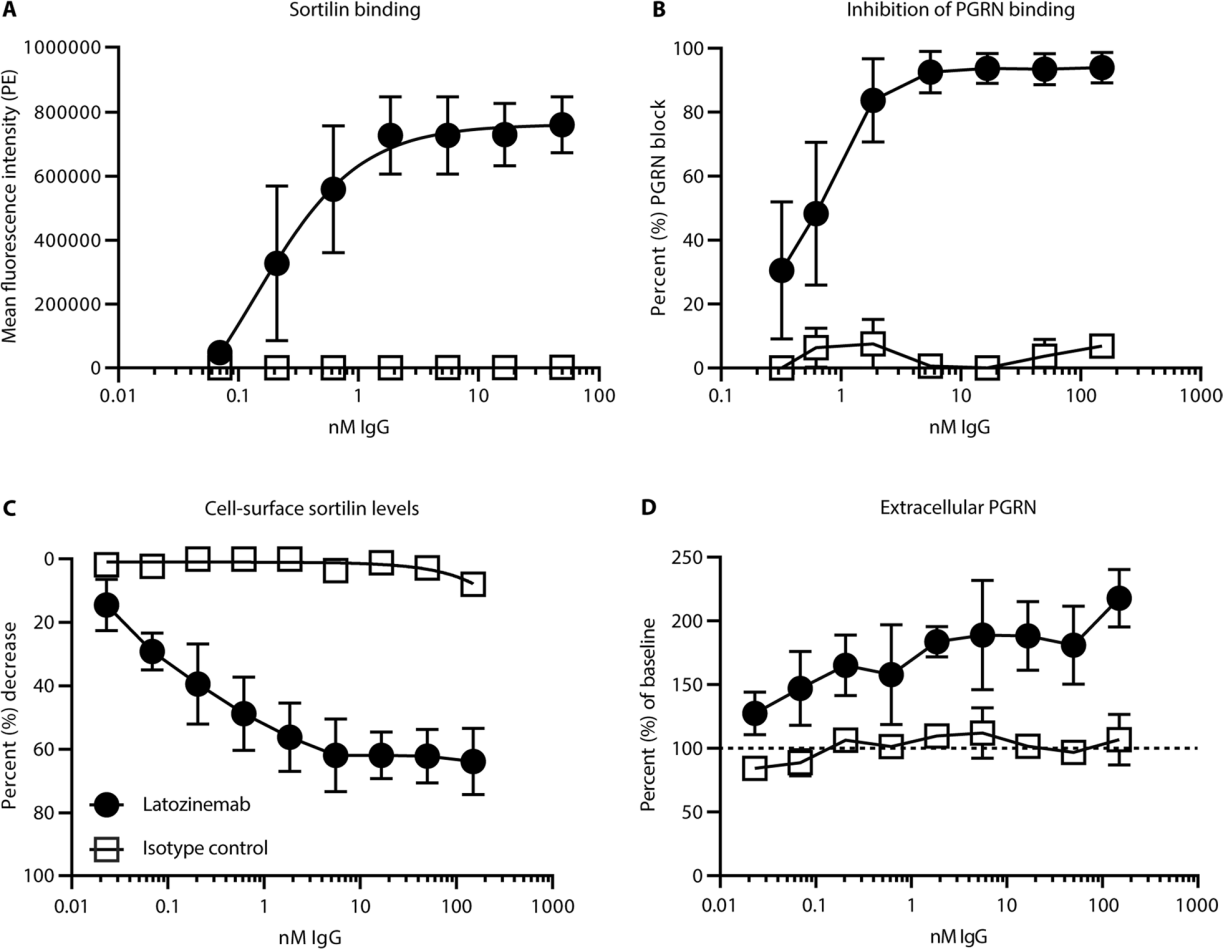
In vitro characterization of latozinemab. **A** Binding curve of sortilin-expressing HEK293T cells by latozinemab. n = 4 technical replicates for latozinemab, n = 5 technical replicates for isotype control. **B** Percentage of dose-dependent blocking of human PGRN binding to sortilin by latozinemab. n = 3 technical replicates for latozinemab, n = 2 technical replicates for isotype control. **C** Dose-dependent decrease in surface sortilin expression on U251 cells. n = 3 technical replicates for latozinemab, n = 2 technical replicates for isotype control. **D** Extracellular PGRN is increased relative to baseline (dashed line) in the presence of latozinemab. n = 3 technical replicates for latozinemab, n = 2 technical replicates for isotype control. All data represent the mean ± SD. 2–5 technical replicates shown for each experiment, each experiment was repeated N > 3, representative data are shown. PE, phycoerythrin; PGRN, progranulin

An HEK293T cell line stably transfected with human sortilin showed a binding curve (K_D_) of 0.13 ± 0.029 nM for latozinemab (Fig. 1A), while no binding occurred on untransfected HEK293T cells (data not shown). Cell-based competition assay with biotinylated human PGRN in the presence or absence of latozinemab further demonstrated a dose-dependent blockage of biotinylated human PGRN binding (Fig. 1B).

We next determined whether latozinemab alters the endogenous levels of sortilin in human U251 cells. Latozinemab was found to reduce cell surface expression of sortilin in a dose-dependent manner, whereas cells treated with control isotype showed no appreciable change in sortilin expression (Fig. 1C). The maximal decrease in sortilin cell surface expression by latozinemab at 150 nM was 63.8%, with a half-maximal effective concentration of 0.105 ± 0.018 nM. Based on the calculated mean differences and standard deviations of the treatment groups at all doses tested, the percent decrease in sortilin cell surface levels from baseline was at least 30% more in the samples treated with latozinemab at 0.206 nM or higher doses, as compared to their respective isotype controls. To look for parallel changes in PGRN levels, PGRN was quantified from the supernatants of the treated cells. Latozinemab elicited a dose-dependent increase of PGRN of up to 2.17-fold (Fig. 1D). The mean differences and standard deviations for each dose were compared and demonstrated that extracellular PGRN levels were at least 30% higher from baseline with latozinemab at 0.023 nM or higher doses, as compared to isotype controls.

### The murine cross-reactive anti-sortilin antibody S15JG increases PGRN levels in vivo and rescues a behavioral deficit in a mouse model of FTD-*GRN*

**Fig. 2:**
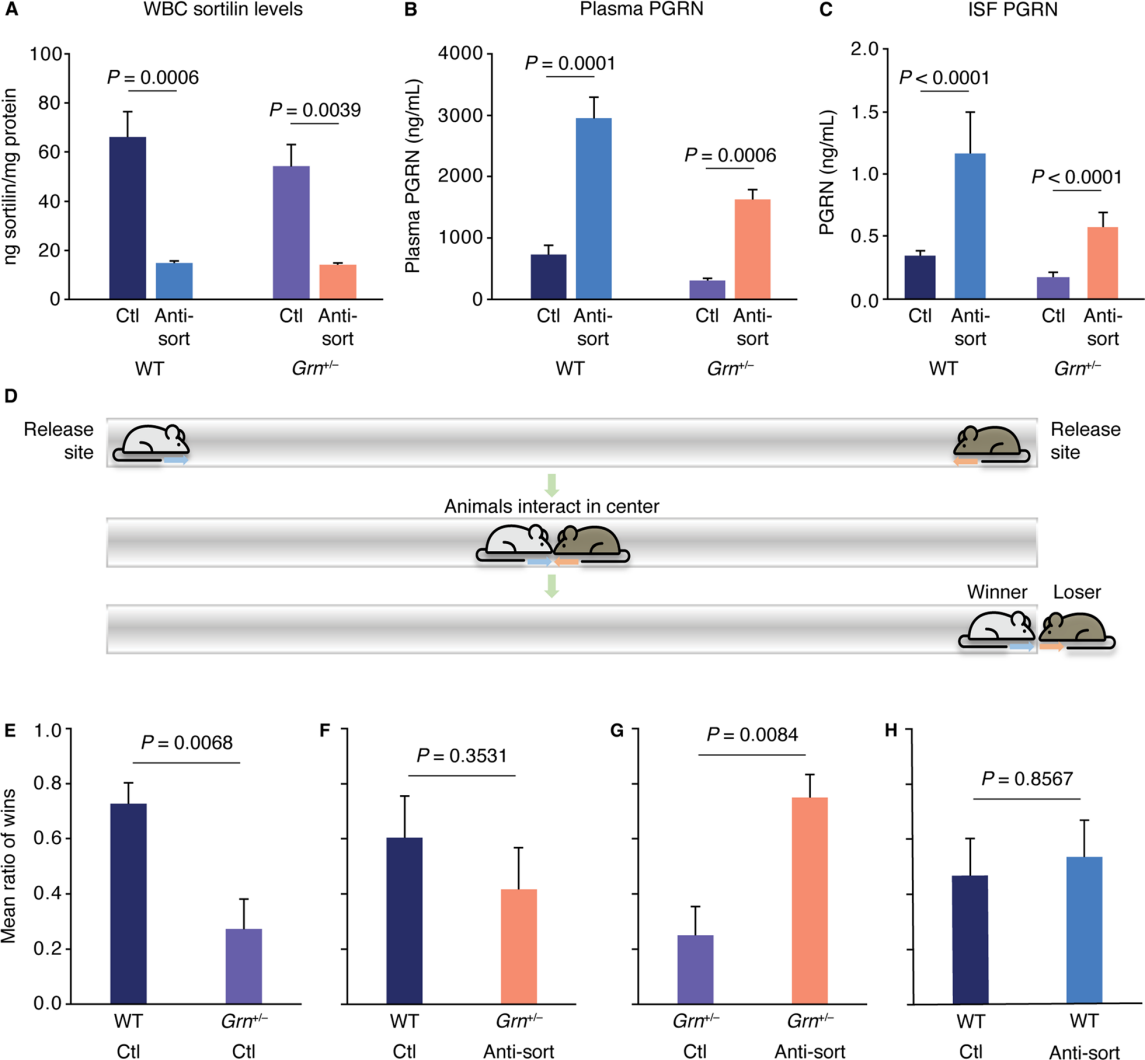
In vivo assessment of an anti-sortilin antibody in a mouse model of *GRN* haploinsufficiency. **A** Cell lysate sortilin levels and **B** plasma PGRN levels from WT and *Grn*^+/−^ mice that received weekly i.p. injections of S15JG or isotype control for 4.5 weeks. **C** PGRN levels measured from ISF 33–48 h post injection demonstrated a significant main effect of antibody treatment (*P* = 0.0089) by Mann–Whitney *U* tests. (A-C) N = 4–6 per group. All data represent the mean ± SEM. *P* values are by planned post hoc Tukey’s tests and are adjusted for multiple comparisons. **D** Schematic of social dominance test. **E–H** Ratio of the number of matches won by each animal in a given matchup. Male and female pairs were tested at 19–21 months of age (n = 9–12 per group). *P* values are by Mann–Whitney *U* tests. All data represent the mean ± SEM. Anti-sort, anti-sortilin antibody S15JG; Ctl, control; ISF, interstitial fluid; PGRN, progranulin

To determine the target engagement and efficacy of S15JG, a mouse cross-reactive antibody that partly blocks the interaction of PGRN with sortilin and strongly decreases sortilin levels, WT and *Grn*^+/−^ mice were used to model the PGRN haploinsufficiency that occurs in FTD-*GRN* [49]. Repeated injections of S15JG significantly reduced expression of sortilin in lysates from WBCs isolated from WT (77.6% reduction) and *Grn*^+/−^ (74% reduction) mice relative to their control-treated counterparts (Fig. 2A). The levels of PGRN in control-treated *Grn*^+/−^ mice were 50% lower than control-treated WTs, as expected (Fig. 2B) [49]. The S15JG–induced decrease in sortilin levels (Fig. 2A) was accompanied by a 3- to fivefold elevation in plasma PGRN levels in both WT and *Grn*^+/−^ mice, with PGRN being restored to above-normal levels in the S15JG–treated *Grn*^+/−^ mice (Fig. 2B). To evaluate PGRN levels in the CNS, microdialysis sampling was used to measure PGRN in the ISF following a single injection of 40-mg/kg S15JG. PGRN levels in the ISF from S15JG–treated mice of both genotypes were ~ 3 times that of their control-treated counterparts (Fig. 2C), suggesting that anti-sortilin treatment can successfully restore PGRN in the brains of *Grn*^+/−^ animals back to physiological levels.

*Grn*^+/−^ mice have been shown to display a behavioral phenotype in a social dominance assay [45]. In this assay (Fig. 2D), 2 mice are placed in a tube facing each other, and 1 of them must retreat for the mice to be able to exit the tube. The mouse that retreats is deemed the “loser.” Aged *Grn*^+/−^ mice lose the majority of these matches when paired against WT controls, but restoring neuronal PGRN in these mice is sufficient to reverse the behavioral phenotype [44, 45].

To determine if S15JG can rescue this behavioral phenotype, 21-month-old *Grn*^+/−^ mice and WT littermates were first tested to confirm the presence of the behavioral phenotype. These mice were then treated with weekly i.p. injections of 40 mg/kg S15JG or an isotype control antibody for 4.5 weeks and then retested. As expected [44, 45], control-treated *Grn*^+/−^ mice lost 70% to 80% of matches against control-treated WT littermates (Fig. 2E). However, when S15JG–treated *Grn*^+/−^ mice were tested against control-treated WT littermates, the win rate increased to approximately 40% to 50% (Fig. 2F), with a loss of a statistically significant difference between the groups. This trend suggests that restoration of PGRN back to physiological levels in the aged mice can rescue the behavioral phenotype associated with PGRN haploinsufficiency that was observed in control-treated mice. We further tested S15JG–treated *Grn*^+/−^ mice against control-treated *Grn*^+/−^ mice and found that the S15JG–treated *Grn*^+/−^ mice behaved similarly to control-treated WT mice and won 70% to 80% of the matches against control-treated *Grn*^+/−^ mice (Fig. 2G). Moreover, control-treated WT mice won similar numbers of matches against S15JG–treated WT mice (Fig. 2H), suggesting that S15JG does not lead to hyperactivity, aggression, or any other detectible adverse behavior in this test when PGRN levels are elevated above normal.

### Latozinemab decreases sortilin levels in WBCs and increases PGRN levels in plasma and CSF of cynomolgus monkeys

**Fig. 3:**
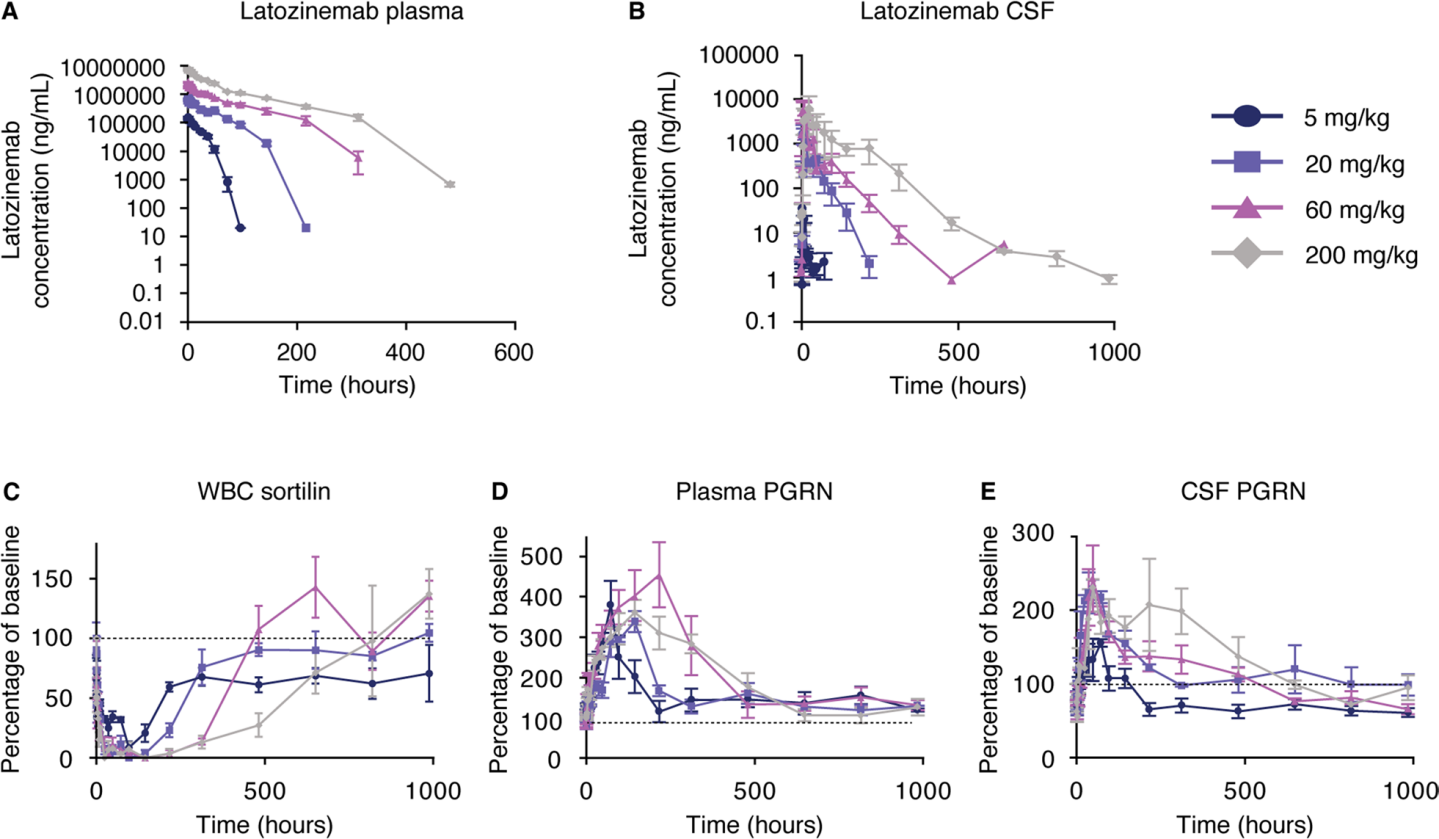
Latozinemab decreases sortilin levels in WBCs and increases PGRN levels in plasma and CSF of cynomolgus monkeys. **A–B** Plasma **A** and CSF **B** concentrations of latozinemab antibody as a function of time. **C** Sortilin concentrations measured as a function of time in WBCs. **D–E** Plasma **D** and CSF **E** PGRN levels measured as a function of time. Male monkeys, 24–50 months of age, were used (n = 3 per group). All data represent the mean ± SEM. Dashed lines in C-E indicate baseline. CSF, cerebrospinal fluid; PGRN, progranulin

To guide dosing parameters for a clinical trial, we next assessed the PK characteristics of varying doses of latozinemab in cynomolgus monkeys for up to 41 days after a single injection. For plasma (Fig. 3A and Additional file 1: Table S1) and CSF (Fig. 3B and Additional file 1: Table S2) latozinemab measurements, the maximum observed concentration (C_max_) and area under the concentration time curve (AUC) (including AUC from time 0 to time t and AUC from time 0 to infinity) increased as the dose was increased. CSF latozinemab concentrations were approximately 0.1% of those measured in plasma, as expected for CNS penetration of monoclonal antibodies [50].

Sortilin levels were decreased in isolated WBC lysates by all doses of latozinemab (Fig. 3C). The 60- and 200-mg/kg doses resulted in an earlier and longer-sustained decrease in sortilin levels compared with the 5- and 20-mg/kg doses (Additional file 1: Tables S3–S4). Across doses, sortilin levels were significantly decreased from baseline for 144–480 h post injection (Additional file 1: Table S3).

Maximal elevations of plasma PGRN levels across all doses of latozinemab were up to fourfold higher than baseline (Fig. 3D). The latozinemab-induced increase in plasma PGRN occurred as early as 6 h post injection for all doses but reached statistical significance at 24 h post injection (Additional file 1: Table S5). Though the C_max_ for plasma latozinemab measurements increased as the dose increased (Additional file 1: Table S1), the peak increase in plasma PGRN levels was similar for all doses (Fig. 3D and Additional file 1: Table S6). However, the PGRN levels remained elevated above baseline for longer durations with the higher latozinemab doses (Fig. 3D and Additional file 1: Table S6). We also quantified CSF PGRN concentrations and found that latozinemab treatment resulted in a 2- to threefold increase in CSF PGRN at the peak of the response (Fig. 3E). Although the 5-mg/kg dose did not increase CSF PGRN above baseline levels, all other doses led to a significant increase in CSF PGRN within 12–24 h post injection (Additional file 1: Table S7). The duration of the latozinemab-induced increase lasted longer with increased doses (Fig. 3E and Additional file 1: Tables S7–S8).

### A single infusion of latozinemab in HVs and aFTD-*GRN* participants decreases WBC sortilin and increases plasma and CSF PGRN

The results of the preclinical pharmacology, PK, and PD studies prompted the start of clinical trials. AL001-1 (NCT03636204) was a first-in-human phase 1 study designed to evaluate the safety, tolerability, PK, and PD of latozinemab. In the first part of the AL001-1 study, latozinemab was administered via i.v. infusion to HVs in a SAD design, including 2-, 6-, 15-, 30-, and 60-mg/kg doses and placebo. In the second part of the study, latozinemab was administered as a single dose of 60 mg/kg to aFTD-*GRN* participants that had 30% to 50% of the normal levels of PGRN. For PK endpoints, serum and CSF concentrations of latozinemab were measured at specified timepoints before and after infusion. For PD assessments, changes from baseline in the levels of PGRN in plasma and CSF and in sortilin expression in WBCs were quantified.

#### Participants

A total of 56 participants were enrolled in the study, including 50 HVs who were randomly assigned to the SAD groups and 6 aFTD-*GRN* participants (Table S9). All participants received a dose of study drug or placebo.

Baseline demographics and characteristics were generally similar across treatment groups for the HVs and aFTD-*GRN* participants (Additional file 1: Table S10). Overall, the age of participants ranged from 19 to 71 years. More males were enrolled than females, and most participants were White.

#### Pharmacokinetics

**Fig. 4:**
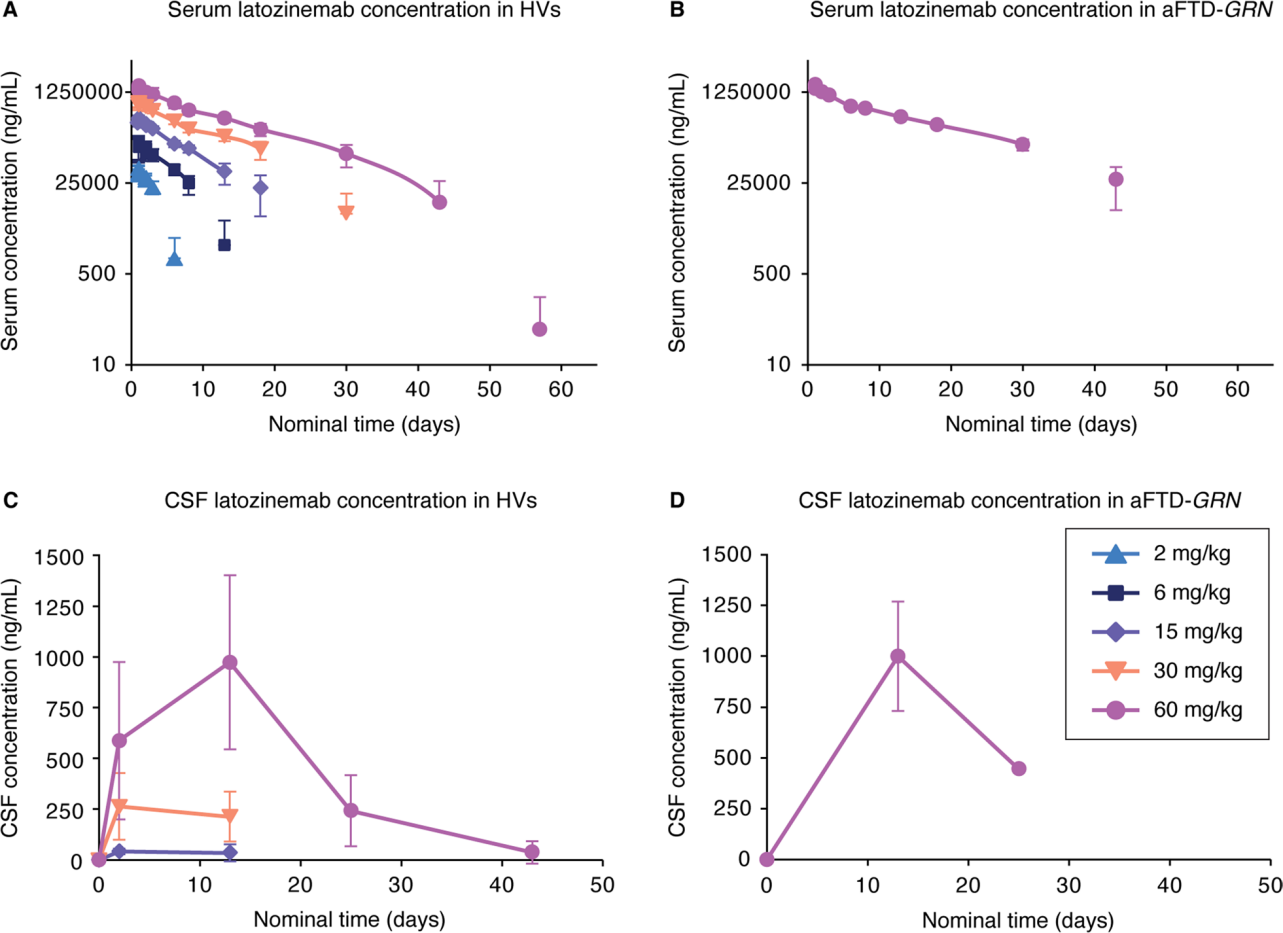
Pharmacokinetics of latozinemab in HVs and aFTD-*GRN* participants. **A–B** Mean ± SD serum concentrations of latozinemab plotted as a function of time for HVs in the SAD groups **A** and aFTD-*GRN* participants who received the 60-mg/kg dose **B**. For the HV SAD groups **A**, n = 4–13 participants/group/timepoint, and for the aFTD*-GRN* group **B**, n = 8 at all timepoints, except for days 15 (n = 7 at 4 h post dose), 85 (n = 4), and 141 (n = 3). **C–D** Mean ± SD CSF concentrations of latozinemab plotted as a function of time for HVs in the SAD groups **C** and aFTD-*GRN* participants **D**; note that CSF samples were not collected for HVs in the 2-mg/kg and 6-mg/kg dose groups. For the HV SAD groups **C**, n = 6 participants/group/timepoint, except for the 60-mg/kg dose group prior to dosing (n = 13) and on day 13 (n = 5). For the aFTD-*GRN* group **D**, n = 5 at all timepoints, except for day 25 (n = 1). Nominal time after dose is relative to the end of infusion. Placebo-treated subjects were excluded from the PK population. aFTD-*GRN*, asymptomatic carrier of *GRN* mutations causative of frontotemporal dementia; CSF, cerebrospinal fluid; HV, healthy volunteer

Following a single infusion of latozinemab at 2–60 mg/kg in HVs, the overall shape of the mean serum concentration–time profiles for latozinemab were similar for each dose group, exhibiting mean peak exposures on day 1 (Fig. 4A). Measurable concentrations of latozinemab were seen for 30 days or longer in groups that received 30 mg/kg or greater. A similar serum concentration–time profile was seen following a single infusion in aFTD-*GRN* participants (Fig. 4B).

For HVs in the SAD groups, the mean CSF concentrations of latozinemab appeared to increase in a dose-dependent manner, with the highest levels observed by day 3 for the 15- and 30-mg/kg dose groups and by day 13 for the 60-mg/kg dose group (Fig. 4C). After reaching the maximum measured CSF concentrations of latozinemab, concentrations declined and approached levels near the lower limit of detection by day 42 for the 60-mg/kg dose group. CSF levels of latozinemab in aFTD-*GRN* participants were similar to those seen in HVs receiving the same dose (Fig. 4D). CSF:serum partition coefficients were less than 1% (Additional file 1: Table S11).

Serum PK parameters demonstrated that mean total and peak exposures of latozinemab increased with increasing dose level in the SAD groups (Additional file 1: Table S12), with approximately dose-proportional increased C_max_ whereas AUC values increased in a greater than proportional manner (Table S13). C_max_ values of latozinemab were attained ~ 1–3 h post dose for the HV SAD groups and ~ 1.5 h post dose for aFTD-*GRN* participants. PK parameters in aFTD-*GRN* participants were similar to those observed in HVs who received the same dose. In the HVs, clearance of latozinemab decreased with increasing doses of latozinemab, and the volume of distribution remained between 2.08 L and 3.15 L (Additional file 1: Table S12).

#### Pharmacodynamics

**Fig. 5:**
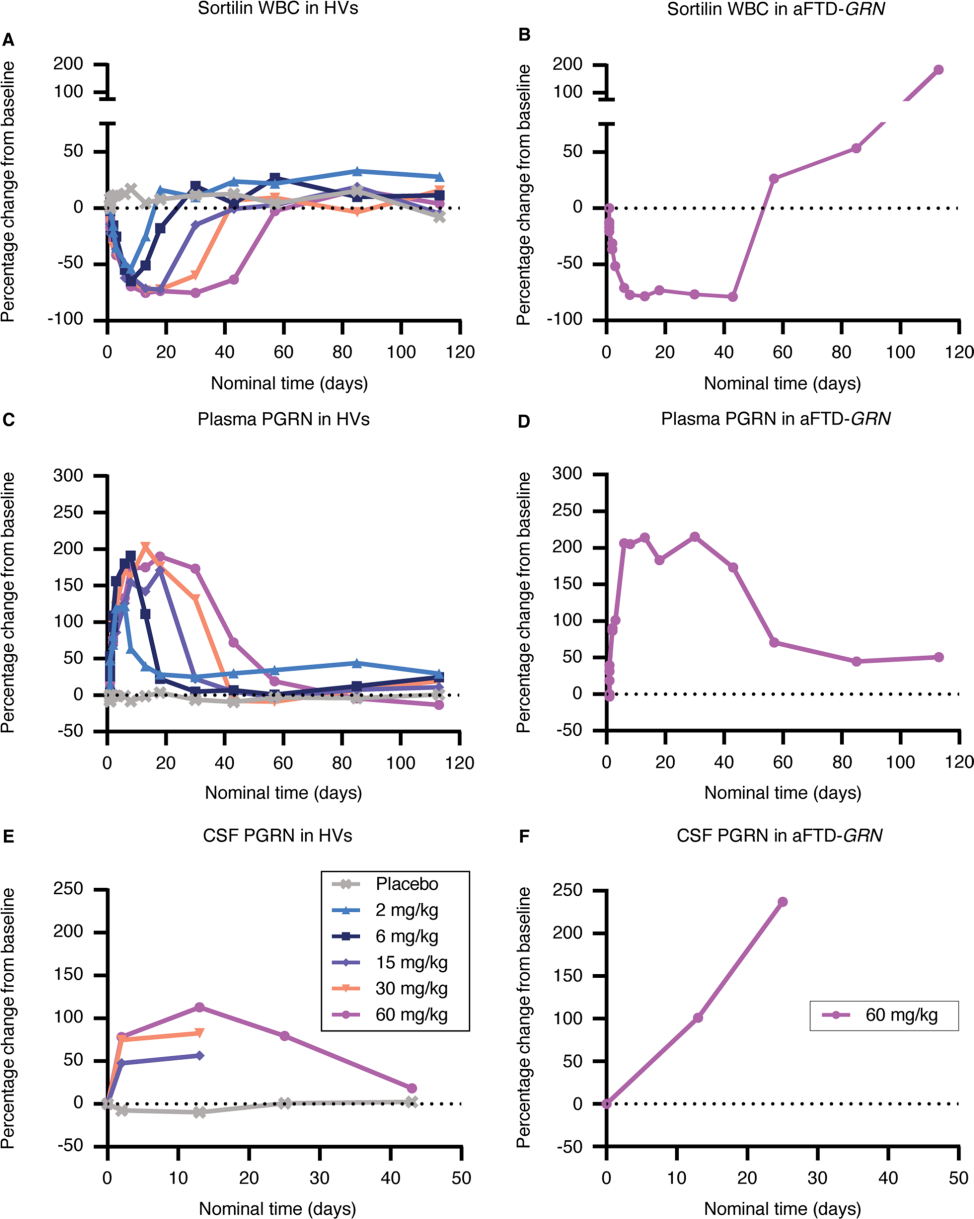
Latozinemab decreases sortilin in WBCs and increases PGRN levels in the plasma and CSF of HVs and aFTD-*GRN* participants. **A–B** Median percentage change from baseline in sortilin in WBCs from HVs who were administered a SAD of latozinemab **A** and from aFTD-*GRN* participants who received a single injection of 60-mg/kg latozinemab **B**. For the HV SAD groups **A**, n = 4–13 participants/group/timepoint and for the aFTD-*GRN* group **B**, n = 3–5 participants/timepont. **C–D** Median percentage change from baseline in PGRN levels in plasma from HVs **C** and from the aFTD-*GRN* group **D**. For the HV SAD groups **C**, n = 4–13 participants/group/timepoint and for the aFTD-*GRN* group **D**, n = 5 at all timepoints, except on days 2 (30 h post dose), 57, 85, and 113 (n = 3) **E–F** Median percent change from baseline in PGRN levels in CSF from HVs **E** and from the aFTD-*GRN* group **F**; note that CSF samples were not collected for HVs in the 2-mg/kg and 6-mg/kg dose groups. For the HV SAD groups **E**, n = 2–13 participants/group/timepoint and for the aFTD-*GRN* group **F**, n = 5 at baseline and day 13 and n = 1 at day 25. Nominal timepoints are relative to the end of infusion. The percent change from baseline was calculated for each individual based on their own predose value. Dashed lines indicate baseline. aFTD-*GRN*, asymptomatic carriers of *GRN* mutations causative of frontotemporal dementia; HV, healthy volunteer; PGRN, progranulin

To assess the effect of latozinemab on sortilin levels, we quantified the concentration of sortilin and total protein in human lysed WBCs. The percentage change from baseline in WBC sortilin levels decreased following a single administration of latozinemab in both HVs and aFTD-*GRN* participants (Fig. 5A, B, Additional file 1: Fig. S2, and Additional file 1: Table S14). The decrease in sortilin appeared to be dose-dependent in the HV SAD groups, with a more robust and longer lasting decrease observed in participants who received the higher doses (Fig. 5A). An apparent increase in sortilin WBC levels (relative to baseline values) was observed after day 57, at which time there was no concomitant decrease in PGRN, but this was driven by 2 individuals who had abnormally low baseline sortilin values; these individuals’ post-treatment absolute sortilin levels were similar to those of other participants (Additional file 1: Fig. S1). Thus, we do not believe there was a continuous increase in sortilin WBC levels 50 + days after treatment and that the sortilin results are consistent with the PGRN results.

Latozinemab administration resulted in an increase in plasma PGRN levels in the HVs (Fig. 5C), with the maximum percentage change from baseline in each of the SAD groups being significantly greater than that in the placebo-treated group (Additional file 1: Fig. S2 and Additional file 1: Table S15). Unlike the dose-dependent effect that was observed for peak serum latozinemab concentrations (Fig. 4A), the peak increase in plasma PGRN levels was comparable across doses (Fig. 5C). The increase in plasma PGRN that occurred in aFTD-*GRN* participants (Fig. 5D) was similar to that observed in HVs who received the same latozinemab dose. In addition, latozinemab increased CSF PGRN levels in both HVs and aFTD-*GRN* participants (Fig. 5E, F), with an apparent dose-dependent increase in the HVs. The relative increase from baseline in CSF PGRN levels in the 15-, 30-, and 60-mg/kg dose groups was significantly greater than in the placebo group on day 13 (Additional file 1: Fig. S2 and Additional file 1: Table S16).

#### Safety and tolerability

Overall, 32 of 50 HVs (64%) reported a total of 70 treatment-emergent adverse events (TEAEs), with similar rates of TEAEs reported for participants treated with latozinemab (63.2%) or placebo (66.7%; Table 1). The majority of TEAEs were mild or moderate in severity and deemed not related to the study drug. Two HVs experienced an SAE (rhabdomyolysis), neither of which was considered related to the study drug; 1 case (in which the individual received placebo) was attributed to strenuous exercise and the other case (in which the individual received 60 mg/kg latozinemab) was suspected to be due to acute alcohol intoxication and recovery. No DLAEs or TEAEs led to study discontinuation in the HVs. The most frequently reported (≥ 10%) TEAEs (Table 2) were post–lumbar puncture syndrome (n = 9; 18%) and puncture-site pain (n = 5; 10%). TEAEs generally occurred at a similar or lower incidence for participants in the latozinemab dose groups compared with the pooled placebo group, with no treatment- or dose-related trends observed for the frequency of TEAEs.

**Table 1:** Summary of TEAEs (safety population)

	Double-blind, SAD HVs								Open-label
		Latozinemab dose level							aFTD-*GRN* 60 mg/kg (N = 6)
	Pooled placebo (n = 12)	2 mg/kg (n = 7)	6 mg/kg (n = 6)	15 mg/kg (n = 6)	30 mg/kg (n = 6)	60 mg/kg (n = 13)	Total AL001 (n = 38)	Total (N = 50)	
Any TEAE, n (%) [E]	8 (66.7)	2 (28.6)	5 (83.3)	4 (66.7)	5 (83.3)	8 (61.5)	24 (63.2)	32 (64.0) [70]	4 (66.7)
Any treatment-related TEAE, n (%) [E]	1 (8.3)^a^	0 (0.0)	0 (0.0)	0 (0.0)	1 (16.7)^a^	0 (0.0)	1 (2.6)	2 (4.0)	1 (16.7)^b^
Severity of TEAEs, n (%) [E]									
Mild	0 (0.0)	0 (0.0)	2 (33.3)	1 (16.7)	0 (0.0)	3 (23.1)	6 (15.8)	6 (12.0)	4 (66.7)
Moderate	7 (58.3)	2 (28.6)	3 (50.0)	3 (50.0)	5 (83.3)	4 (30.8)	17 (44.7)	24 (48.0)	0 (0.0)
Severe	0 (0.0)	0 (0.0)	0 (0.0)	0 (0.0)	0 (0.0)	0 (0.0)	0 (0.0)	0 (0.0)	0 (0.0)
Life-threatening	1 (8.3)	0 (0.0)	0 (0.0)	0 (0.0)	0 (0.0)	1 (7.7)	1 (2.6)	2 (4.0)	0 (0.0)
Death	0 (0.0)	0 (0.0)	0 (0.0)	0 (0.0)	0 (0.0)	0 (0.0)	0 (0.0)	0 (0.0)	0 (0.0)
Severity of treatment-related TEAEs, n (%) [E]									
Mild	1 (8.3)	0 (0.0)	0 (0.0)	0 (0.0)	1 (16.7)	0 (0.0)	1 (2.6)	2 (4.0)	1 (16.7)
Moderate	0 (0.0)	0 (0.0)	0 (0.0)	0 (0.0)	0 (0.0)	0 (0.0)	0 (0.0)	0 (0.0)	0 (0.0)
Severe	0 (0.0)	0 (0.0)	0 (0.0)	0 (0.0)	0 (0.0)	0 (0.0)	0 (0.0)	0 (0.0)	0 (0.0)
Life-threatening	0 (0.0)	0 (0.0)	0 (0.0)	0 (0.0)	0 (0.0)	0 (0.0)	0 (0.0)	0 (0.0)	0 (0.0)
Death	0 (0.0)	0 (0.0)	0 (0.0)	0 (0.0)	0 (0.0)	0 (0.0)	0 (0.0)	0 (0.0)	0 (0.0)
Any SAE, n (%) [E]	1 (8.3)^c^	0 (0.0)	0 (0.0)	0 (0.0)	0 (0.0)	1 (7.7)^c^	1 (2.6)	2 (4.0)	0 (0.0)
Any treatment-related SAE, n (%) [E]	0 (0.0)	0 (0.0)	0 (0.0)	0 (0.0)	0 (0.0)	0 (0.0)	0 (0.0)	0 (0.0)	0 (0.0)
Any TEAE leading to discontinuation, n (%) [E]	0 (0.0)	0 (0.0)	0 (0.0)	0 (0.0)	0 (0.0)	0 (0.0)	0 (0.0)	0 (0.0)	0 (0.0)
Any DLAE, n (%) [E]	0 (0.0)	0 (0.0)	0 (0.0)	0 (0.0)	0 (0.0)	0 (0.0)	0 (0.0)	0 (0.0)	NA

aFTD-*GRN*, asymptomatic carrier of *GRN* mutations causative of frontotemporal dementia; DLAE, dose-limiting adverse event; HV, healthy volunteer; SAD, single ascending dose; TEAE, treatment-emergent adverse event; SAE, serious adverse event

^a^ Post–lumbar puncture syndrome

^b^ Myalgia, lipase increased, and tachycardia

^c^ Rhabdomyolysis

**Table 2:** TEAEs occurring in ≥ 5% participants overall

	Double-blind, single-ascending-dose healthy volunteers								Open-label
		Latozinemab dose level							aFTD-*GRN* 60 mg/kg (N = 6)
MedDRA preferred term n (%) [E]	Pooled placebo (n = 12)	2 mg/kg (n = 7)	6 mg/kg (n = 6)	15 mg/kg (n = 6)	30 mg/kg (n = 6)	60 mg/kg (n = 13)	Total AL001 (n = 38)	Total (N = 50)	
Post–lumbar puncture syndrome	2 (16.7)	0	0	1 (16.7)	3 (50.0)	3 (23.1)	7 (18.4)	9 (18.0)	1 (16.7)
Puncture site pain	2 (16.7)	0	0	0	0	3 (23.1)	3 (7.9)	5 (10.0)	0
Headache	0	0	0	0	3 (50.0)	1 (7.7)	4 (10.5)	4 (8.0)	0
Anemia	1 (8.3)	0	0	1 (16.7)	1 (16.7)	0	2 (5.3)	3 (6.0)	0
Vomiting	0	1 (14.3)	0	0	1 (16.7)	1 (7.7)	3 (7.9)	3 (6.0)	0

aFTD-*GRN*, asymptomatic carrier of *GRN* mutations causative of frontotemporal dementia; E, number of events reported; MedDRA, medical dictionary for regulatory activities; TEAE, treatment-emergent adverse event

Four aFTD-*GRN* participants (66.7%) reported a total of 10 TEAEs (Table 1). All TEAEs in this group were mild in severity and the majority were not related to the study drug. One aFTD-*GRN* participant experienced 3 treatment-related TEAEs (myalgia, lipase increased, and tachycardia). No aFTD-*GRN* participants experienced an SAE or a TEAE leading to early discontinuation. No individual TEAEs were reported by more than 1 participant in the aFTD-*GRN* group.

Overall, no apparent differences or trends were observed among the groups in mean changes from baseline for hematology and coagulation, clinical chemistry, or urinalysis. Several participants experienced clinical laboratory abnormalities, with no notable treatment- or dose-related trends and only 1 clinical abnormality deemed possibly related to treatment (increased lipase in 1 aFTD-*GRN* participant). Vital sign measurements throughout the study were generally similar to those observed at baseline, and no treatment or dose-related trends in ECGs were observed. One aFTD-*GRN* participant experienced mild treatment-related tachycardia; no other vital sign TEAEs were considered treatment related.

## Discussion

PGRN haploinsufficiency, as a result of a LOF *GRN* mutation, is a cause of FTD (FTD-*GRN*). Advances in human genetics helped to identify sortilin as a receptor for PGRN endocytosis and degradation, which is the primary determinant of PGRN levels. These advances suggest that therapeutic efforts to treat FTD-*GRN* could target the sortilin-PGRN axis to prevent PGRN degradation and thereby elevate its level in the brains of individuals with FTD-*GRN*. Here, we describe the mechanism of action and target engagement of latozinemab, a monoclonal antibody that blocks sortilin-PGRN interactions and targets the sortilin receptor for degradation in cell-based and animal studies. We further evaluated the safety profile of latozinemab and demonstrated its target engagement in a first-in-human phase 1 clinical trial with HVs and aFTD-*GRN* participants.

As proof of principle, we performed in vitro cell-based assays and found that latozinemab effectively binds sortilin with high affinity, blocks the interaction between PGRN and sortilin, and decreases sortilin levels on the cell surface. PGRN is a secreted lysosomal chaperone, neuronal survival factor, and immune regulatory molecule, and none of these functions have been shown to be affected by the absence of sortilin. First, genetic ablation of *Sort1* leads to chronic elevation of PGRN in the brain and serum [25], but does not prevent PGRN from entering the lysosomes through multiple alternative receptors [29], or from promoting neuronal survival [51]. Second, genetic ablation of PGRN is associated with lysosomal abnormalities, inflammation, and neurodegeneration [52], whereas genetic ablation of *Sort1* is not associated with such changes, and was in fact reported to protect mice from age- and injury-dependent neuronal degeneration [53]. Third, PGRN that was engineered not to bind to sortilin is functional and capable of replacing endogenous PGRN in vivo [54]. Fourth, people with genetic variations that affect *SORT1* levels were not reported to display lysosomal abnormalities or neurodegeneration [24], unlike those with LOF mutations in *GRN*. PGRN was shown to still localize to the lysosome in the absence of sortilin through a pathway involving interactions with prosaposin, M6PR, and LRP1 [29, 55]. Although the evidence indicates that loss of sortilin does not contribute to lysosomal abnormalities, the role of PGRN on lysosomal function is crucial. Therefore, the effects of latozinemab on lysosomal biomarkers will be assessed in a phase 2 clinical trial in FTD-*GRN* patients (NCT03987295).

While PGRN plays an important role in lysosomal function, the full-length protein has also been shown to have important neurotrophic and immunosuppressive effects. Additionally, the protein is cleaved into 7 individual granulins and a paragranulin whose ranges of activity have not been fully elucidated [56–58]. However, there is evidence that granulins have an important function in lysosomal activity. Granulin E and multi-granulin peptides were shown to bind cathepsin D (CTSD) [59], an important lysosomal protease responsible for degradation of various substrates. Granulin E has also been shown to enhance the conversion of pro-CTSD to mature CTSD, and to increase the activity of mature CTSD [60, 61]. DeMuynk and colleagues [62] also showed that granulin E can mediate the neurotrophic effects of PGRN, independent of its binding to sortilin. Although this provides evidence that PGRN and granulin E can function independently of sortilin, the study was limited in that it did not evaluate the impact of reduced sortilin on individual granulins, a point that can be addressed in future studies. This and other work assessing the role of individual granulin peptides in FTD-*GRN* may lead to a better mechanistic understanding of PGRN’s role in lysosomal function and neuronal protection. Beyond its protective role for neuronal and microglial function, PGRN has been implicated in tumorigenesis, but a causal link between increased PGRN and cancer is not clear. There are diverse signaling pathways affected by PGRN, which alone may not be necessary for tumorigenesis. Blocking the interaction between PGRN and sortilin was able to prevent lung metastasis and infiltration of cancer cells [63]. Moreover, the levels of PGRN in latozinemab-treated FTD-*GRN* participants were similar to the levels observed in untreated, healthy individuals, which suggests that this potential risk of latozinemab in FTD-*GRN* patients is low.

Here, we have shown that latozinemab or the mouse cross-reactive S15JG were able to cause a robust decrease in cell surface and total sortilin levels as well as a parallel increase in PGRN levels in vivo. It is notable that we found increased PGRN levels across species in both the plasma and brain biofluid compartments post treatment (ISF via microdialysis in rodents and CSF from primates and human). Treatment of aged *Grn*^+/–^ mice for 1 month with S15JG reversed a behavioral phenotype associated with PGRN haploinsufficiency in these animals. Although this behavioral phenotype is apparent in the Grn^+/–^ mice , other dysfunction or pathology such as glial activation or accumulation of lipofuscin are not well defined. Arrant et al. [45] identified abnormal signaling pathways and neuronal morphology that may explain this phenotype, which was not assessed in this study. Overall, the results suggest that latozinemab can effectively target the CNS and may be able to mitigate the neuropathologic effects of decreased PGRN levels in FTD.

Clinical data were consistent with the preclinical data from rodents and nonhuman primates. Latozinemab was generally well tolerated when administered as a single i.v. dose of 2, 6, 15, 30, or 60 mg/kg to HVs or as a single i.v. dose of 60 mg/kg to aFTD-*GRN* participants. TEAEs were reported at similar rates in the HVs and aFTD-*GRN* participants, and the majority were mild or moderate in severity. No participants experienced a DLAE or TEAE leading to early study discontinuation.

## Conclusions

Latozinemab is being developed for the treatment of carriers of *GRN* mutations causative of FTD to reduce the rate of neurodegeneration by increasing levels of PGRN in the periphery and brain. Here, latozinemab elicited a long-lasting increase in PGRN in the serum and CSF of aFTD-*GRN* participants, and restored PGRN levels to those of HVs. Together, these findings demonstrate that the sortilin receptor is a viable target for PGRN-based therapy, particularly in patients who have PGRN deficiency leading to FTD, and support the continued development of latozinemab. Studies are ongoing to determine if latozinemab-induced elevations in PGRN levels can slow the progression of FTD-associated neurodegeneration (NCT04374136).

## Supplementary Information

**Additional file 1: Figure S1.** Individual WBC concentrations of sortilin. **Figure S2.** Comparison of changes from baseline in PD endpoints between placebo and SAD groups. **Table S1.** Estimated half-life and PK parameters of latozinemab antibody in plasma following a single i.v. injection of 5, 20, 60, or 200 mg/kg in cynomolgus monkeys. **Table S2.** Estimated half-life and PK parameters of latozinemab antibody in CSF following a single i.v. injection of 5, 20, 60, or 200 mg/kg in cynomolgus monkeys. **Table S3.** *P* values of decreased WBC sortilin levels over baseline as a function of time. **Table S4.** *P* values of normalized plasma sortilin for between-group comparisons. **Table S5.** *P* values of normalized plasma PGRN levels relative to baseline. **Table S6.** *P* values of normalized plasma PGRN for between-group comparisons. **Table S7.** *P* values of normalized CSF PGRN levels relative to baseline. **Table S8.** *P* values of normalized CSF PGRN for between-group comparisons. **Table S9.** Subject disposition. **Table S10.** Baseline demographics and characteristics. **Table S11.** Summary of CSF concentrations partition coefficient of latozinemab. **Table S12.** Geometric meanserum PK parameters of latozinemab in HVs and aFTD-*GRN* participants. **Table S13.** Statistical assessment of dose proportionality for latozinemab. **Table S14.** Statistical comparison of maximum percentage change from baseline in WBC sortilin. **Table S15.** Statistical comparison of maximum percentage change from baseline in plasma PGRN. **Table S16.** Statistical comparison of relative percentage change from baseline in CSF PGRN.

## Data Availability

All data are available in the main text or supplementary materials.

## Abbreviations

ACK: Ammonium-chloride-potassium
ADCC: Antibody-dependent cell-mediated cytotoxicity
AE: Adverse event
aFTD-*GRN*: Asymptomatic *GRN* mutation carriers
APC: Allophycocyanin
AUC: Area under the concentration time curve
BLQ: Below the limit of quantification
BSA: Bovine serum albumin
*C9orf72*: Chromosome 9 open reading frame 72
C_max_: Maximum observed concentration
CNS: Central nervous system
CSF: Cerebrospinal fluid
CTSD: Cathepsin D
DLAE: Dose-limiting adverse event
ECG: Electrocardiogram
ELISA: Enzyme-linked immunosorbent assay
FACS: Fluorescence-activated cell sorting
FBS: Fetal bovine serum
FTD: Frontotemporal dementia
*GRN*: Progranulin gene
HRP: Horseradish peroxidase
HV: Healthy volunteer
IEC: Independent ethics committee
IgG: Immunoglobulin
i.p.: Intraperitoneal
IRB: Institutional review board
ISF: Interstitial fluid
i.v.: Intravenous
LLOQ: Lower limit of quantification
LOF: Loss of function
LRP1: Low-density lipoprotein receptor–related protein 1
M6PR: Mannose 6-phosphate receptor
MD: Multiple dose
MedDRA: Medical dictionary for regulatory activities
MFI: Mean fluorescence intensity
MRD: Minimum required dilution
PD: Pharmacodynamics
PE: Phycoerythrin
PGRN: Progranulin
PK: Pharmacokinetics
QC: Quality control
S15JG: Rodent cross-reactive anti-sortilin antibody
SAD: Single ascending dose
SAE: Serious adverse event
*SORT1*: Sortilin gene
TEAE: Treatment-emergent adverse event
TK: Toxicokinetic
TMB: Tetramethylbenzidine
ULOQ: Upper limit of quantification
WBC: White blood cell
WT: Wild type
