# Supplementary material for: Latozinemab, a novel progranulin-elevating therapy for frontotemporal dementia
Source: J Transl Med. 2023 Jun 15;21:387. doi: 10.1186/s12967-023-04251-y (PMC10268535; doi:10.1186/s12967-023-04251-y)
Supplement: Supplementary file 1 — Additional file 1: Figure S1. Individual WBC concentrations of sortilin. Figure S2. Comparison of changes from baseline in PD endpoints between placebo and SAD groups. Table S1. Estimated half-life and PK parameters of latozinemab antibody in plasma following a single i.v. injection of 5, 20, 60, or 200 mg/kg in cynomolgus monkeys. Table S2. Estimated half-life and PK parameters of latozinemab antibody in CSF following a single i.v. injection of 5, 20, 60, or 200 mg/kg in cynomolgus monkeys. Table S3. P values of decreased WBC sortilin levels over baseline as a function of time. Table S4. P values of normalized plasma sortilin for between-group comparisons. Table S5. P values of normalized plasma PGRN levels relative to baseline. Table S6. P values of normalized plasma PGRN for between-group comparisons. Table S7. P values of normalized CSF PGRN levels relative to baseline. Table S8. P values of normalized CSF PGRN for between-group comparisons. Table S9. Subject disposition. Table S10. Baseline demographics and characteristics. Table S11. Summary of CSF concentrations partition coefficient of latozinemab. Table S12. Geometric meanserum PK parameters of latozinemab in HVs and aFTD-GRN participants. Table S13. Statistical assessment of dose proportionality for latozinemab. Table S14. Statistical comparison of maximum percentage change from baseline in WBC sortilin. Table S15. Statistical comparison of maximum percentage change from baseline in plasma PGRN. Table S16. Statistical comparison of relative percentage change from baseline in CSF PGRN. [file 12967_2023_4251_MOESM1_ESM.docx]

**Latozinemab, a novel progranulin-elevating therapy for frontotemporal dementia**

Michael Kurnellas, Ananya Mitra, Tina Schwabe, Robert Paul, Andrew E. Arrant, Erik D. Roberson, Michael Ward, Felix Yeh, Hua Long, Arnon Rosenthal

**Additional Information**

**ADDITIONAL FIGURES**

**
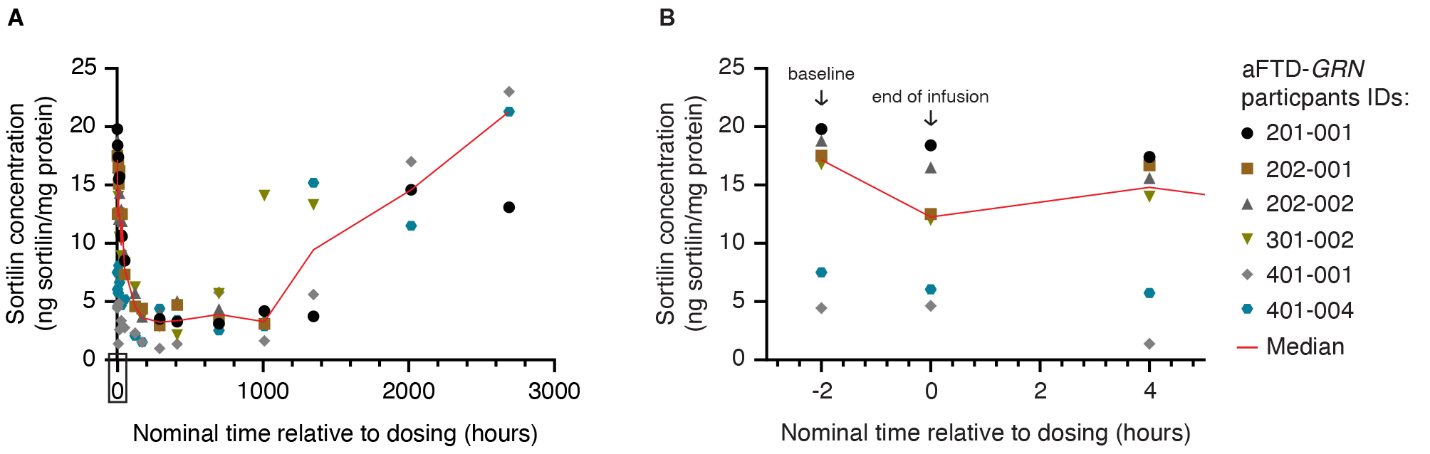
**

**Additional Figure S1. Individual WBC concentrations of sortilin. (A)** Sortilin concentrations in WBCs were measured at various time points from each aFTD-*GRN* participant beginning at baseline (prior to latozinemab infusion) and continuing up to 113 days postdose. The boxed portion of the x-axis indicates the data that are expanded in B. **(B)** Sortilin concentrations in WBCS from aFTD-*GRN* participants that were measured before, immediately after, and within 4 hours of latozinemab infusion.

**
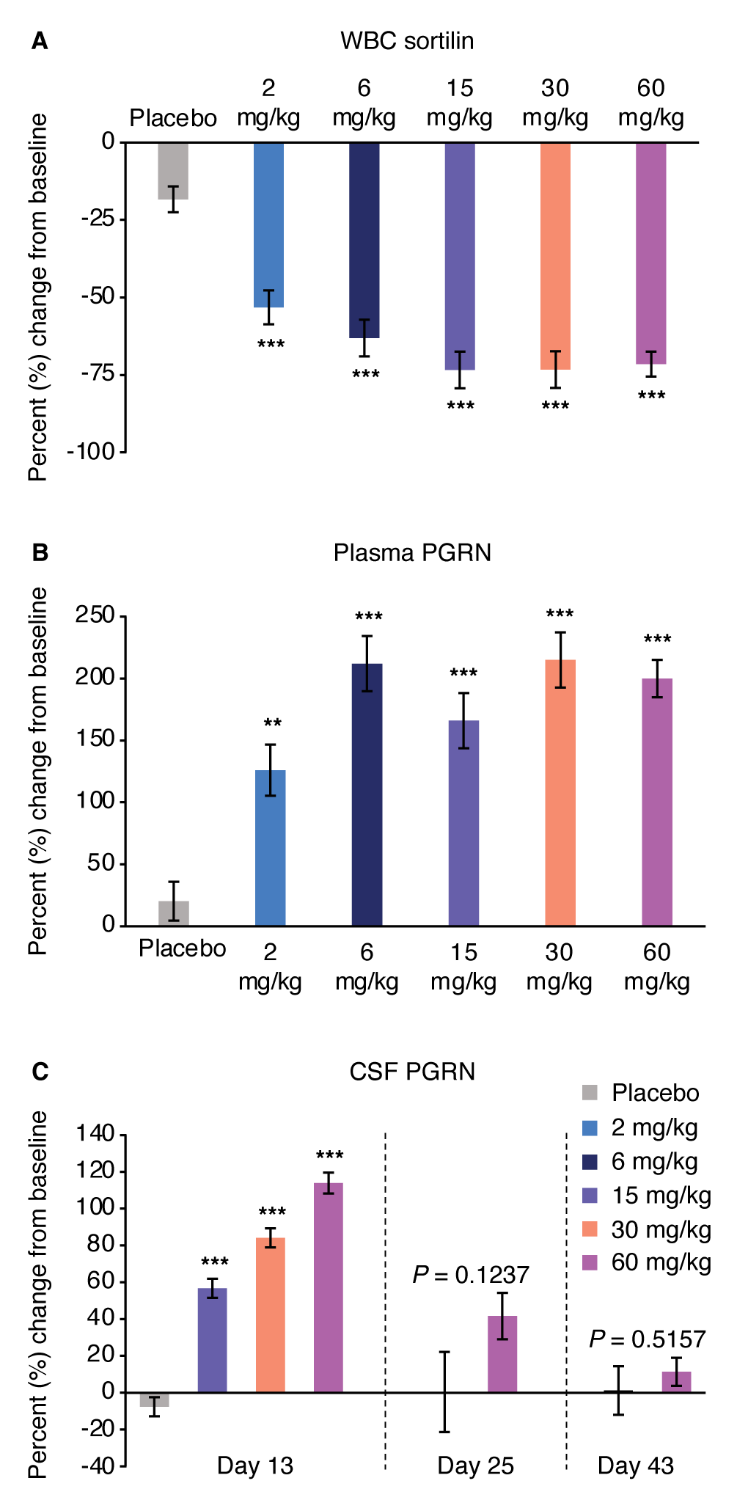
**

**Additional Figure S2. Comparison of changes from baseline in PD endpoints between placebo and SAD groups.** (**A-B**) The maximum absolute percentage change from baseline of WBC sortilin (A) and plasma PGRN (B) over 113 days following a single i.v. administration of latozinemab was compared for each treatment group vs placebo. (**C**) The relative percentage change in CSF PGRN from baseline on days 13, 25, and 43 was compared between HVs who received 15-, 30-, or 60-mg/kg latozinemab and HVs who received placebo. All data represent the least squares mean±SEM.

^**^ *P* < 0.001 by Dunnett’s test adjusted for multiple comparisons.

^***^ *P* < 0.0001 by Dunnett’s test adjusted for multiple comparisons.

CSF, cerebrospinal fluid; HV, healthy volunteer; PD, pharmacodynamic; PGRN, progranulin; SAD, single ascending dose.

**ADDITIONAL TABLES**

| **Dose**  **(mg/kg)** | **C_0_**  **(µg/mL)** | **C_max_**  **(µg/mL)** | **C_max_/dose**  **(µg/mL)/ (mg/kg)** | **AUC_0-t_**  **(µg*hr/mL)** | **AUC_0-inf_**  **(µg*hr/mL)** | **AUC_0-inf_/dose**  **(µg*hr/mL)/ (mg/kg)** | **CL**  **(mL/hr/kg)** | **t_max_**  **(hr)** | **t_1/2_**  **(hr)** | **V_ss_**  **(mL/kg)** |
| --- | --- | --- | --- | --- | --- | --- | --- | --- | --- | --- |
| ^5^ | ^164 (24.7)^ | ^156 (9.69)^ | ^31.2 (1.94)^ | ^2870 (331)^ | ^2870 (331)^ | ^574 (66.2)^ | ^1.76 (0.211)^ | ^0.278 (0.192)^ | ^4.69 (1.14)^ | ^30.9 (0.82)^ |
| ^20^ | ^742 (91.2)^ | ^697 (28.1)^ | ^34.9 (1.4)^ | ^26500 (3000)^ | ^26600 (2820)^ | ^1330 (141)^ | ^0.757 (0.08)^ | ^0.278 (0.192)^ | ^13.3 (7.04)^ | ^32.2 (3.03)^ |
| ^60^ | ^3140 (246)^ | ^2570 (163)^ | ^42.9 (2.72)^ | ^118000 (21600)^ | ^119000 (20400)^ | ^1980 (341)^ | ^0.515 (0.0961)^ | ^0.167 (0)^ | ^42 (6.69)^ | ^3.9 (2.47)^ |
| ^200^ | ^7980 (1600)^ | ^7910 (1230)^ | ^39 (6.13)^ | ^376000 (19300)^ | ^379000 (19300)^ | ^1890 (96.5)^ | ^0.529 (0.0265)^ | ^0.278 (0.192)^ | ^47.3 (8.99)^ | ^42 (3.5)^ |

**Additional Table S1. Estimated half-life and PK parameters of latozinemab antibody in plasma following a single i.v. injection of 5, 20, 60, or 200 mg/kg in cynomolgus monkeys.** The antibody PK parameters were estimated using noncompartmental analysis. The C_max_ and AUC increased congruent with the escalation in the dose given. The half-life for the plasma concentration of AL001 was 4.7 hours for 5 mg/kg, 13.3 hours for 20 mg/kg, 42 hours for 60 mg/kg, and 47.3 hours for 200 mg/kg. All data represent group means (±SD).

AUC, area under the concentration time curve; AUC_0-inf_, area under the concentration time curve from hour 0 to infinity for day 1; AUC_0-t_, area under the concentration time curve from hour 0 to the last measurable concentration; C_0_, back-extrapolated concentration at time 0; CL, systemic clearance; C_max_, maximum plasma concentration; PK, pharmacokinetic; t_1/2_, elimination half-life; t_max_, time of maximum observed concentration; V_SS,_ volume of distribution at steady state.

| **Dose**  **(mg/kg)** | **C_max_**  **(ng/mL)** | **C_max_/dose**  **(ng/mL)/(mg/kg)** | **AUC_0-t_**  **(ng*hr/mL)** | **AUC_0-inf_**  **(ng*hr/mL)** | **AUC_0-inf_/dose**  **(ng*hr/mL)/(mg/kg)** | **t_max_**  **(hr)** | **t_1/2_**  **(hr)** |
| --- | --- | --- | --- | --- | --- | --- | --- |
| 5 | 27 (7.67) | 5.4 (1.53) | 179 (54.1) | 203 (65.6) | 40.6 (13.1) | 5.5 (4.33) | 17.8 (7.61) |
| 20 | 2240 (1340) | 112 (66.8) | 38100 (24000) | 38200 (24000) | 1910 (1200) | 8.67 (3.06) | 23.8 (3.71) |
| 60 | 6780 (5470) | 113 (91.1) | 122000 (58600) | 122000 (58500) | 2040 (975) | 16.7 (16.8) | 41.6 (10.3) |
| 200 | 4600 (6000) | 23 (30) | 395000 (359000) | 395000 (359000) | 1970 (1800) | 36 (12) | 118 (82.1) |

**Additional Table S2. Estimated half-life and PK parameters of latozinemab antibody in CSF following a single i.v. injection of 5, 20, 60, or 200 mg/kg in cynomolgus monkeys.** The amount of antibody measured in the CSF and its duration as measured by C_max_ and AUC were increased as the amount of antibody injected was increased. CSF latozinemab antibody concentration was on average 0.1% the amount measured in plasma. All data represent group means (±SD).

AUC, area under the concentration time curve; AUC_0-inf_, area under the concentration time curve from hour 0 to infinity for day 1; AUC_0-t_, area under the concentration time curve from hour 0 to the last measurable concentration; C_max_, maximum observed concentration; CSF, cerebrospinal fluid; PK, pharmacokinetic;; t_1/2_, elimination half-life; t_max_, time of maximum observed concentration.

| **Time (hr)** | **5 mg/kg** | **20 mg/kg** | **60 mg/kg** | **200 mg/kg** |
| --- | --- | --- | --- | --- |
| 0.16 | ns | ns | 0.0354 | 0.0026 |
| 0.5 | 0.0122 | ns | 0.0001 | 0.0094 |
| 1 | ns | 0.0018 | ns | 0.024 |
| 2 | ns | ns | ns | 0.0073 |
| 4 | 0.0005 | ns | 0.0083 | 0.0003 |
| 6 | 0.0001 | 0.0001 | 0.001 | 0.0001 |
| 8 | 0.0005 | 0.0018 | 0.0001 | 0.0001 |
| 12 | 0.0027 | 0.0001 | 0.0001 | 0.0001 |
| 24 | 0.0001 | 0.0001 | 0.0001 | 0.0001 |
| 36 | 0.0001 | 0.0001 | 0.0001 | 0.0001 |
| 48 | 0.0001 | 0.0001 | 0.0001 | 0.0001 |
| 72 | 0.0001 | 0.0001 | 0.0001 | 0.0001 |
| 96 | 0.0001 | 0.0001 | 0.0001 | 0.0001 |
| 144 | 0.0001 | 0.0001 | 0.0001 | 0.0001 |
| 216 | ns | 0.0001 | 0.0001 | 0.0001 |
| 312 | ns | ns | 0.0001 | 0.0001 |
| 480 | ns | ns | ns | 0.0001 |
| 648 | ns | ns | ns | ns |
| 816 | ns | ns | ns | ns |
| 984 | ns | ns | ns | ns |

**Additional Table S3. *P* values of decreased WBC sortilin levels over baseline as a function of time.** Data were analyzed via 2-way ANOVA with Dunnett’s multiple comparisons test. All doses resulted in significant decreases in WBC sortilin levels from baseline. The effect of latozinemab on WBC sortilin had an earlier onset and lasted longer with the higher doses.

|  | **0.167 hr** | **0.5 hr** | **216 hr** | **312 hr** | **480 hr** | **648 hr** | **984 hr** |
| --- | --- | --- | --- | --- | --- | --- | --- |
| 5 mg/kg vs 20 mg/kg | ns | ns | ns | ns | ns | ns | ns |
| 5 mg/kg vs 60 mg/kg | ns | ns | 0.0015 | 0.0023 | 0.0128 | <0.0001 | 00002 |
| 5 mg/kg vs 200 mg/kg | ns | ns | 0.0017 | 0.0021 | ns | ns | 0.0001 |
| 20 mg/kg vs 60 mg/kg | ns | 0.0313 | ns | 0.0003 | ns | 0.0033 | ns |
| 20 mg/kg vs 200 mg/kg | 0.019 | ns | ns | 0.0003 | 0.0003 | ns | ns |
| 60 mg/kg vs 200 mg/kg | ns | ns | ns | ns | <0.0001 | <0.0001 | ns |

**Additional Table S4. *P* values of normalized plasma sortilin for between-group comparisons.** Data were analyzed via 2-way ANOVA with Tukey multiple comparisons test.

| **Time (hr)** | **5 mg/kg** | **20 mg/kg** | **60 mg/kg** | **200 mg/kg** |
| --- | --- | --- | --- | --- |
| 6 | ns | ns | 0.0447 | ns |
| 8 | ns | ns | ns | ns |
| 12 | ns | ns | 0.0197 | 0.0001 |
| 24 | 0.0006 | ns | 0.0015 | 0.0001 |
| 36 | 0.0001 | ns | 0.0001 | 0.0001 |
| 48 | 0.0001 | ns | 0.0001 | 0.0001 |
| 72 | 0.0001 | 0.0001 | 0.0001 | 0.0001 |
| 96 | 0.0001 | 0.0001 | 0.0001 | 0.0001 |
| 144 | 0.0084 | 0.0001 | 0.0001 | 0.0001 |
| 216 | ns | ns | 0.0001 | 0.0001 |
| 312 | ns | ns | 0.0001 | 0.0001 |

**Additional Table S5. *P* values of normalized plasma PGRN levels relative to baseline.** Data were analyzed via 2-way ANOVA with Dunnett’s multiple comparisons test. All doses resulted in an increase in PGRN levels as early as 6 hours post injection but reached statistical significance for most groups by 24 hours post injection. PGRN levels remained significantly increased for the longest duration in the 60- and 200-mg/kg groups.

PGRN, progranulin.

|  | **36 hr** | **48 hr** | **96 hr** | **144 hr** | **216 hr** | **312 hr** |
| --- | --- | --- | --- | --- | --- | --- |
| 5 mg/kg vs 20 mg/kg | ns | 0.0109 | ns | 0.0012 | ns | ns |
| 5 mg/kg vs 60 mg/kg | ns | ns | 0.0042 | <0.0001 | <0.0001 | 0.0014 |
| 5 mg/kg vs 200 mg/kg | ns | ns | ns | <0.0001 | <0.0001 | 0.0009 |
| 20 mg/kg vs 60 mg/kg | 0.0158 | 0.0007 | ns | ns | <0.0001 | 0.0003 |
| 20 mg/kg vs 200 mg/kg | ns | 0.0247 | ns | ns | 0.0006 | 0.0002 |
| 60 mg/kg vs 200 mg/kg | ns | ns | ns | ns | 0.0005 | ns |

**Additional Table S6. *P* values of normalized plasma PGRN for between-group comparisons.** Data were analyzed via 2-way ANOVA with Tukey multiple comparisons test.

PGRN, progranulin.

| **Time (hr)** | **5 mg/kg** | **20 mg/kg** | **60 mg/kg** | **200 mg/kg** |
| --- | --- | --- | --- | --- |
| 12 | ns | 0.0001 | ns | ns |
| 24 | ns | 0.0001 | 0.0001 | 0.0026 |
| 36 | ns | mv | 0.0001 | 0.0001 |
| 48 | mv | mv | 0.0004 | mv |
| 72 | ns | 0.484 | 0.0305 | mv |
| 96 | mv | ns | mv | mv |
| 144 | ns | ns | ns | 0.0001 |
| 216 | ns | ns | ns | 0.0005 |

**Additional Table S7. *P* values of normalized CSF PGRN levels relative to baseline.** Data were analyzed via 2-way ANOVA with Dunnett’s multiple comparisons test.

CSF, cerebrospinal fluid; mv, missing value (Dunnett’s multiple comparison test could not be performed due to missing CSF sample at given time point); PGRN, progranulin.

|  | **12 hr** | **24 hr** | **36 hr** | **72 hr** | **96 hr** | **144 hr** | **216 hr** | **312 hr** |
| --- | --- | --- | --- | --- | --- | --- | --- | --- |
| 5 mg/kg vs  20 mg/kg | 0.0009 | 0.0011 | 0.0007 | ns | mv | ns | ns | ns |
| 5 mg/kg vs  60 mg/kg | ns | 0.0029 | <0.0001 | 0.0462 | mv | 0.0165 | ns | ns |
| 5 mg/kg vs  200 mg/kg | ns | ns | 0.0005 | 0.003 | mv | <0.0001 | <0.0001 | 0.0122 |
| 20 mg/kg vs  60 mg/kg | ns | ns | mv | ns | mv | ns | ns | ns |
| 20 mg/kg vs  200 mg/kg | 0.0011 | ns | mv | mv | mv | 0.0028 | 0.0003 | ns |
| 60 mg/kg vs  200 mg/kg | ns | ns | ns | mv | mv | 0.0217 | 0.036 | ns |

**Additional Table S8. *P* values of normalized CSF PGRN for between-group comparisons.** Data were analyzed via 2-way ANOVA with Tukey multiple comparisons test.

CSF, cerebrospinal fluid; mv, missing value (Tukey multiple comparison test could not be performed due to missing CSF sample at given time point); PGRN, progranulin.

|  |  | **Double-blind, SAD HVs** | | | | | | **Open-label** |
| --- | --- | --- | --- | --- | --- | --- | --- | --- |
|  |  |  | **Latozinemab dose level** | | | | |  |
|  | **Total**  **(N = 56)** | **Pooled placebo**  **(n = 12)** | **2 mg/kg**  **(n = 7)** | **6 mg/kg**  **(n = 6)** | **15 mg/kg**  **(n = 6)** | **30 mg/kg**  **(n = 6)** | **60 mg/kg**  **(n = 13)** | **aFTD-*GRN***  **60 mg/kg**  **(n = 6)^a^** |
| **Total number of participants, n (%)**  **Completed study drug**  **Discontinued study drug**  **Completed study**  **Discontinued study** | 56 (100.0)  0  39 (69.6)  17 (30.4) | 12 (100.0)  0  9 (75.0)  3 (25.0) | 7 (100.0)  0  4 (57.1)  3 (42.9) | 6 (100.0)  0  5 (83.3)  1 (16.7) | 6 (100.0)  0  6 (100.0)  0 | 6 (100.0)  0  5 (83.3)  1 (16.7) | 13 (100.0)  0  7 (53.8)  6 (46.2) | 6 (100.0)  0  3 (50.0)  3 (50.0) |
| **Primary reason for discontinuation, n (%)**  **Adverse event**  **Lost to follow-up**  **Investigator discretion**  **Protocol deviation**  **Withdrawal by participant**  **Use of nonpermitted concomitant drug**  **Participation in another clinical trial**  **Loss of ability to freely provide consent**  **Occurrence of clinically relevant exclusion criterion**  **Transferred to phase 2 AL001-2 protocol**  **Other** | 0  6 (10.7)  1 (1.8)  0  6 (10.7)  0  0  0  0  3 (5.4)  1 (1.8) | 0  1 (8.3)  0  0  2 (16.7)  0  0  0  0  0  0 | 0  2 (28.6)  0  0  1 (14.3)  0  0  0  0  0  0 | 0  0  1 (16.7)  0  0  0  0  0  0  0  0 | 0  0  0  0  0  0  0  0  0  0  0 | 0  0  0  0  1 (16.7)  0  0  0  0  0  0 | 0  3 (23.1)  0  0  2 (15.4)  0  0  0  0  0  1 (7.7) | 0  0  0  0  0  0  0  0  0  3 (50.0)  0 |
| **Analysis populations, n (%)**  **Safety population^b^**  **PK population^c^**  **PD population^d^** | 56 (100.0)  44 (78.6)  56 (100.0) | 12 (100.0)  0  12 (100.0) | 7 (100.0)  7 (100.0)  7 (100.0) | 6 (100.0)  6 (100.0)  6 (100.0) | 6 (100.0)  6 (100.0)  6 (100.0) | 6 (100.0)  6 (100.0)  6 (1000) | 13 (100.0)  13 (100.0)  13 (100.0) | 6 (100.0)  6 (100.0)  6 (100.0) |

**Additional Table S9. Subject disposition (enrolled population).**

^a^ One aFTD-*GRN* patient was incorrectly dosed at 30 mg/kg.

^b^ The safety population included all enrolled participants who received at least 1 dose of study drug (latozinemab or placebo).

^c^ The PK population included all participants in the safety population who had adequate assessments for determination of PK parameters.

^d^ The PD population included all participants in the safety population who had both a baseline and at least 1 postdose PD assessment.

aFTD-*GRN*, asymptomatic carrier of *GRN* mutations causative of frontotemporal dementia; HVs, healthy volunteers; PD, pharmacodynamic; PK, pharmacokinetic; SAD, single ascending dose.

|  | **Double-blind, SAD HVs** | | | | | | **Open-label** |
| --- | --- | --- | --- | --- | --- | --- | --- |
|  |  | **Latozinemab dose level** | | | | |  |
|  | **Pooled placebo**  **(n = 12)** | **2 mg/kg**  **(n = 7)** | **6 mg/kg**  **(n = 6)** | **15 mg/kg**  **(n = 6)** | **30 mg/kg**  **(n = 6)** | **60 mg/kg**  **(n = 13)** | **aFTD-*GRN***  **60 mg/kg**  **(n = 6)** |
| **Age (years)**  **Mean (SD)**  **Min, max** | 34.6 (10.00)  23, 53 | 53.7 (6.75)  44, 62 | 40.0 (10.77)  27, 55 | 39.3 (16.03)  23, 64 | 32.5 (13.26)  19, 58 | 41.0 (12.59)  21, 58 | 50.3 (13.69)  47, 71 |
| **Sex, n (%)**  **Female**  **Male** | 4 (33.3)  8 (66.7) | 5 (71.4)  2 (28.6) | 1 (16.7)  5 (83.3) | 3 (50.0)  3 (50.0) | 1 (16.7)  5 (83.3) | 7 (53.8)  6 (46.2) | 1 (16.7)  5 (83.3) |
| **Race, n (%)**  **White**  **Black or African American**  **American Indian or Alaska Native** | 6 (50.0)  5 (41.7)  1 (8.3) | 5 (71.4)  2 (28.0)  0 (0.0) | 3 (50.0)  3 (50.0)  0 | 1 (16.7)  5 (83.3)  0 | 3 (50.0)  3 (50.0)  0 | 6 (46.2)  7 (53.8)  0 | 6 (100.0)  0  0 |
| **Ethnicity, n (%)**  **Hispanic or Latino**  **Not Hispanic or Latino** | 5 (41.7)  7 (58.3) | 0  7 (100.0) | 3 (50.0)  3 (50.0) | 0  6 (100.0) | 1 (16.7)  5 (83.3) | 2 (15.4)  11 (84.6) | 0  6 (100.0) |
| **Screening height (cm), mean (SD)** | 167.20 (6.165) | 167.33 (7.969) | 178.83 (9.026) | 170.80 (4.863) | 174.17 (10.614) | 167.25 (10.959) | 156.58 (45.452) |
| **Screening weight (kg), mean (SD)** | 75.42 (9.813) | 69.91 (13.364) | 89.25 (11.228) | 81.85 (16.693) | 82.43 (12.634) | 74.76 (12.652) | 84.55 (9.092) |
| **Screening BMI (kg/m^2^), mean (SD)** | 26.91 (2.513) | 24.77 (3.122) | 27.83 (2.386) | 27.93 (4.682) | 27.13 (3.092) | 26.59 (2.488) | 28.20 (2.977) |

**Additional Table S10. Baseline demographics and characteristics (safety population).**

aFTD-*GRN*, asymptomatic carriers of *GRN* mutations causative of frontotemporal dementia; HVs, healthy volunteers; SAD, single ascending dose.

| **Treatment** | **Day^a^** | **Nominal time (hr) after dose^b^** | **n** | **Mean** | **SD** | **CV** | **Median** | **Minimum** | **Maximum** |
| --- | --- | --- | --- | --- | --- | --- | --- | --- | --- |
| HV 15 mg/kg (N = 6) | 2  13 | 30  288 | 6  6 | 0.000140  0.000655 | 0.0000377  0.000734 | 27.0  112 | 0.000140  0.000563 | 0.0000868  0 | 0.000190  0.00159 |
| HV 30 mg/kg (N = 6) | 2  13 | 30  288 | 6  6 | 0.000439  0.00124 | 0.000320  0.000917 | 72.8  74.0 | 0.000369  0.000897 | 0.000109  0.000677 | 0.000950  0.00309 |
| HV 60 mg/kg (N = 12) | 2  13  43 | 30  288  1008 | 6  5  5 | 0.000545  0.00270  0.00163 | 0.000398  0.00147  0.00254 | 73.1  54.6  155 | 0.000457  0.00234  0 | 0.000135  0.000965  0 | 0.00109  0.00440  0.00577 |
| aFTD-*GRN* 60 mg/kg  (N = 5) | 13 | 288 | 5 | 0.00236 | 0.000778 | 32.9 | 0.00248 | 0.00137 | 0.00347 |

**Additional Table S11. Summary of CSF concentrations partition coefficient of latozinemab (PK population).** Partition coefficient was calculated as concentration of latozinemab in CSF/latozinemab in serum. Placebo-treated participants were excluded from the PK population.

^a^ Day is relative to the start of the study.

^b^ Nominal time after dose is relative to the end of infusion.

aFTD-*GRN*, asymptomatic carriers of *GRN* mutations causative of frontotemporal dementia; CSF, cerebrospinal fluid; CV, coefficient of variation; HV, healthy volunteer; PK, pharmacokinetic.

|  | **Latozinemab dose levels** | | | | | |
| --- | --- | --- | --- | --- | --- | --- |
|  | **HVs** | | | | | **aFTD-*GRN*** |
| **Parameter** | **2 mg/kg**  **(N = 7)** | **6 mg/kg**  **(N = 6)** | **15 mg/kg**  **(N = 6)** | **30 mg/kg**  **(N = 6)** | **60 mg/kg**  **(N = 12)** | **60 mg/kg**  **(N = 5)** |
| AUC_0-tlast_ (h*µg/mL) | 2090 (40.2) | 11800 (21.2) | 49300 (10.3) | 125000 (20.1) | 310000 (15.3) | 361000 (10.4) |
| AUC_0-inf_ (h*µg/mL) | 2640 (23.1) | 12900 (20.6) | 52100 (12.8) | 134000 (14.6) | 327000 (14.9) | 370000 (12.1) |
| C_max_ (µg/mL) | 46.8 (14.4) | 128 (35.5) | 409 (12.2) | 819 (18.1) | 1640 (18.4) | 1770 (5.75) |
| t_max_ (h)^a^ | 1.08 (1.08, 5.07) | 3.13 (1.07, 49.08) | 1.09 (1.08, 5.15) | 1.05 (1.00, 5.05) | 1.17 (1.05, 49.08) | 1.48 (1.33, 8.92) |
| t_1/2_ (h)^b^ | 29.6 (32.8) | 50.5 (29.5) | 86.4 (28.6) | 119 (23.9) | 164 (22.0) | 188 (19.8) |
| CL (mL/h) | 51.5 (28.6) | 40.6 (33.0) | 23.0 (13.0) | 18.0 (14.6) | 13.4 (24.1) | 14.3 (11.5) |
| V_z_ (L) | 2.08 (54.2) | 2.84 (55.7) | 2.78 (24.7) | 3.00 (33.8) | 3.15 (31.1) | 3.82 (8.90) |
| V_ss_ (L) | 2.23 (47.7) | 3.35 (66.7) | 2.79 (18.5) | 3.20 (20.9) | 3.24 (23.8) | 3.86 (1.45) |
| DNAUC_0-tlast_ ([h*µg/mL]/[mg/kg]) | 1040 (40.2) | 1970 (21.2) | 32.90 (10.3) | 4160 (20.1) | 5160 (15.3) | 6020 (10.4) |
| DNAUC_0-inf_ ([h*µg/mL]/[mg/kg]) | 1320 (23.1) | 2150 (20.6) | 3470 (12.8) | 4470 (14.6) | 5450 (14.9) | 6160 (12.1) |
| DNC_max_ ([µg/mL]/[mg/kg]) | 23.4 (14.4) | 21.4 (35.5) | 27.3 (12.2) | 27.3 (18.1) | 27.4 (18.4) | 29.5 (5.75) |

**Additional Table S12. Geometric mean (CV) serum PK parameters of latozinemab in HVs and aFTD-*GRN* participants (PK population).** Mean exposure levels and half-life values increased with increasing doses of latozinemab. Serum PK parameters following 60 mg/kg i.v. infusion in aFTD-*GRN* patients were similar to HV data at the same dose level.

^a^ For t_max_, the median (minimum, maximum) values are presented.

^b^ For t_1/2_, the mean (CV) values are presented.

aFTD-*GRN*, asymptomatic carriers of *GRN* mutations causative of frontotemporal dementia; AUC_0-inf_, area under the concentration-time curve from time 0 extrapolated to infinity; AUC_0-tlast_, area under the concentration-time curve from time 0 to the last measurable time point; CL, clearance; C_max_, maximum concentration; CV, coefficient of variation; DNAUC_0-inf_, assigned dose (mg/kg) normalized area under the concentration-time curve from time 0 extrapolated to infinity; DNAUC_0-tlast_, assigned dose (mg/kg) normalized area under the concentration-time curve from time 0 to the last measurable time point; DNC_max_, assigned dose (mg/kg) normalized maximum concentration; HV, healthy volunteer; PK, pharmacokinetic; t_max_, time of maximum concentration; t_1/2_, terminal elimination half-life; V_ss_, apparent volume of distribution associated at steady state; V_z_, volume of distribution during the terminal phase.

|  | **Exponent of the power model** | | |  |  |
| --- | --- | --- | --- | --- | --- |
| **Parameter** | **Estimate** | **Lower 90% CI** | **Upper 90% CI** | **Intercept** | **Lack-of-fit *P* value** |
| AUC_0-tlast_ (h*µg/mL) | 1.47 | 1.41 | 1.52 | 6.7 | 0.0753 |
| AUC_0-inf_ (h*µg/mL) | 1.42 | 1.38 | 1.46 | 6.93 | 0.1669 |
| C_max_ (µg/mL) | 1.06 | 1.00 | 1.12 | 3.07 | 0.8791 |

**Additional Table S13. Statistical assessment of dose proportionality for latozinemab (PK population).** A power model, ln(parameter) = ln(a) + b × ln(dose), was used to estimate the slope (exponent) and corresponding 90% CI. A separate model with a quadratic term [log(dose)²] was used to test for a systematic departure from linearity (lack of fit) of the power model. Dose proportionality was concluded if the 90% CI of the slope (β) lay entirely within (0.80, 1.20) for the 2- to 60-mg/kg dose range [i.e., (1 + ln(0.5) / ln(r), 1+ ln(2) / ln(r))], where r is the dose range (highest dose/lowest dose). A formal statistical analysis of dose proportionality for serum latozinemab was performed using the power model. Following a single i.v. infusion of latozinemab at 2 mg/kg to 60 mg/kg in HVs, peak systemic exposure to latozinemab (as measured by C_max_) increased in an approximately dose-proportional manner, whereas total systemic exposure (as measured by AUC_0-tlast_ and AUC_0-inf_) increased in a greater than dose-proportional manner; exponents of the power model ranged from 1.06 to 1.47 in all instances.

AUC_0-inf_, area under the concentration-time curve from time 0 extrapolated to infinity; AUC_0-tlast_, area under the concentration-time curve from time 0 to the last measurable time point; C_max_, maximum concentration; HV, healthy volunteer; PK, pharmacokinetic.

| **Comparison**  **(reference − test)** | **LS mean difference** | **Standard error** | **Lower 95% CI** | **Upper 95% CI** | ***P* value** | **Adjusted *P* value** |
| --- | --- | --- | --- | --- | --- | --- |
| Pooled placebo − 2 mg/kg | 34.9 | 6.89 | 21.06 | 48.83 | <0.0001 | <0.0001 |
| Pooled placebo − 6 mg/kg | 44.9 | 7.24 | 30.26 | 59.45 | <0.0001 | <0.0001 |
| Pooled placebo − 15 mg/kg | 55.1 | 7.24 | 40.53 | 69.73 | <0.0001 | <0.0001 |
| Pooled placebo − 30 mg/kg | 55.1 | 7.24 | 40.46 | 69.65 | <0.0001 | <0.0001 |
| Pooled placebo − 60 mg/kg | 53.2 | 5.80 | 41.54 | 64.92 | <0.0001 | <0.0001 |

**Additional Table S14. Statistical comparison of maximum percentage change from baseline in WBC sortilin (PD population).** The maximum absolute percentage change from baseline of sortilin WBCs over 113 days following single-dose i.v. administration of latozinemab was compared for each treatment group vs the pooled placebo group. Data were analyzed via Dunnett’s test.

LS, least squares; PD, pharmacodynamic.

| **Comparison**  **(reference − test)** | **LS mean difference** | **Standard error** | **Lower 95% CI** | **Upper 95% CI** | ***P* value** | **Adjusted *P* value** |
| --- | --- | --- | --- | --- | --- | --- |
| Pooled placebo − 2 mg/kg | −106 | 25.9 | −158.00 | −53.47 | 0.0002 | 0.0009 |
| Pooled placebo − 6 mg/kg | −191 | 27.3 | −246.25 | −136.36 | <0.0001 | <0.0001 |
| Pooled placebo − 15 mg/kg | −146 | 27.3 | −201.14 | −91.25 | <0.0001 | <0.0001 |
| Pooled placebo − 30 mg/kg | −195 | 27.3 | −249.76 | −139.88 | <0.0001 | <0.0001 |
| Pooled placebo − 60 mg/kg | −180 | 21.8 | −223.92 | −135.94 | <0.0001 | <0.0001 |

**Additional Table S15. Statistical comparison of maximum percentage change from baseline in plasma PGRN (PD population).** The maximum absolute percentage change from baseline of plasma PGRN over 113 days following single-dose i.v. administration of latozinemab was compared following each treatment group vs the pooled placebo group. Data were analyzed via Dunnett’s test.

LS, least squares; PD, pharmacodynamic; PGRN, progranulin.

| **Comparison**  **(reference** − **test)** | **LS mean difference** | **Standard error** | **Lower 95% CI** | **Upper 95% CI** | ***P* value** | **Adjusted *P* value** |
| --- | --- | --- | --- | --- | --- | --- |
| Pooled placebo − 15 mg/kg  (day 13) | −64.4 | 7.31 | −79.65 | −49.06 | <0.0001 | <0.0001 |
| Pooled placebo − 30 mg/kg  (day 13) | −91.8 | 7.31 | −107.07 | −76.48 | <0.0001 | <0.0001 |
| Pooled placebo − 60 mg/kg  (day 13) | −121 | 7.66 | −137.27 | −105.18 | <0.0001 | <0.0001 |
| Pooled placebo − 60 mg/kg  (day 25) | −41.3 | 25.2 | −95.31 | 12.78 | 0.1237 | 0.1237 |
| Pooled placebo − 60 mg/kg  (day 43) | −10.2 | 15.2 | −42.85 | 22.52 | 0.5157 | 0.5157 |

**Additional Table S16. Statistical comparison of relative percentage change from baseline in CSF PGRN (PD population).** Percentage change from baseline 13 days after dosing 15, 30, or 60 mg/kg was compared with placebo using Dunnett’s test to determine any difference between latozinemab treatment and placebo. Percentage change from baseline 25 and 43 days after dosing 60 mg/kg was also compared with placebo via Dunnett’s test.

CSF, cerebrospinal fluid; LS, least squares; PD, pharmacodynamic; PGRN, progranulin.
